# Endothelial PDGF-BB/PDGFR-β signaling promotes osteoarthritis by enhancing angiogenesis-dependent abnormal subchondral bone formation

**DOI:** 10.1038/s41413-022-00229-6

**Published:** 2022-08-29

**Authors:** Zhuang Cui, Hangtian Wu, Ye Xiao, Ting Xu, Junjie Jia, Hancheng Lin, Rongmin Lin, Kun Chen, Yihuang Lin, Kaiqun Li, Xiaohu Wu, Changjun Li, Bin Yu

**Affiliations:** 1 Division of Orthopaedics and Traumatology, Department of Orthopaedics, Nanfang Hospital, Southern Medical University, Guangzhou, Guangdong China; 2 Guangdong Provincial Key Laboratory of Bone and Cartilage Regeneration Medicine, Nanfang Hospital, Southern Medical University, Guangzhou, Guangdong China; 3 Department of Endocrinology, Endocrinology Research Center, Xiangya Hospital of Central South University, Changsha, Hunan 410008 China; 4 National Clinical Research Center for Geriatric Disorders, Xiangya Hospital, Changsha, Hunan 410008 China; 5 Department of Sleep Medicine Center, Nanfang Hospital, Southern Medical University, Guangzhou, Guangdong 510515 China

**Keywords:** Pathogenesis, Bone

## Abstract

The mechanisms that coordinate the shift from joint homeostasis to osteoarthritis (OA) remain unknown. No pharmacological intervention can currently prevent the progression of osteoarthritis. Accumulating evidence has shown that subchondral bone deterioration is a primary trigger for overlying cartilage degeneration. We previously found that H-type vessels modulate aberrant subchondral bone formation during the pathogenesis of OA. However, the mechanism responsible for the elevation of H-type vessels in OA is still unclear. Here, we found that PDGFR-β expression, predominantly in the CD31^hi^Emcn^hi^ endothelium, was substantially elevated in subchondral bones from OA patients and rodent OA models. A mouse model of OA with deletion of PDGFR-β in endothelial cells (ECs) exhibited fewer H-type vessels, ameliorated subchondral bone deterioration and alleviated overlying cartilage degeneration. Endothelial PDGFR-β promotes angiogenesis through the formation of the PDGFR-β/talin1/FAK complex. Notably, endothelium-specific inhibition of PDGFR-β by local injection of AAV9 in subchondral bone effectively attenuated the pathogenesis of OA compared with that of the vehicle-treated controls. Based on the results from this study, targeting PDGFR-β is a novel and promising approach for the prevention or early treatment of OA.

## Introduction

Osteoarthritis (OA) is a highly prevalent and degenerative joint disease that leads to chronic pain and physical disability, resulting in a major financial and clinical burden.^1,2^ The current cartilage-centered viewpoint for the pathogenesis of OA is shifting toward a whole joint model.^3–5^ Accumulating studies have revealed that subchondral bone deterioration is a critical trigger for overlying cartilage degeneration, thus inducing mechanical loading changes in articular cartilage that lead to the pathogenesis of OA.^6–9^ Subchondral bone deterioration results from increased and uncoupled bone remodeling.^4,6^ During this process, excessive mesenchymal stem cells (MSCs) are erroneously recruited to either bone marrow or nonbone resorption pits, resulting in abnormal bone formation in subchondral bone. This process results in abnormal bone formation.^6^

Bone formation is coupled with angiogenesis.^10–12^ Capillaries are indispensable during both bone modeling and bone remodeling to spatiotemporally orchestrate the complicated and dynamic communications among bone tissues.^13–15^ A specific vessel subtype, named H-type or CD31^hi^Emcn^hi^ vessels, was recently identified and shown to link vessel formation with bone formation.^16,17^ A significant increase in H-type vessels in subchondral bone has been observed in our previous studies, forming an active feedback loop with MSCs during the onset of OA.^18,19^ However, the underlying mechanism responsible for the elevation of H-type vessels in OA is still unclear. Platelet-derived growth factor-BB (PDGF-BB)/platelet-derived growth factor receptor beta (PDGFR-β) signaling has a well-established role in blood vessel formation.^20,21^ PDGF-BB/PDGFR-β signaling correlates with the stabilization of newly formed vessels, the orchestration of cellular components for osteogenesis, and an increase in vascularity.^21–23^ PDGF-BB secreted by mononuclear preosteoclasts has been shown to maintain normal long bone and cranial bone homeostasis by enhancing H-type vessels.^24–26^ Knockout of PDGF-BB in tartrate-resistant acid phosphatase-positive (TRAP^+^) mononuclear cells impaired angiogenesis-coupled osteogenesis,^24^ whereas bone formation and even fracture healing were enhanced if PDGF-BB expression in TRAP^+^ mononuclear cells was elevated.^27^ Moreover, abnormal overexpression of PDGF-BB in preosteoclasts resulted in vascular and skeletal disorders such as arterial stiffening and OA.^28,29^ Su W et al. recently revealed that excessive secretion of PDGF-BB from preosteoclasts promoted aberrant angiogenesis-dependent bone formation in subchondral bone, leading to OA pathogenesis.^29,30^ Interestingly, endothelial cell (EC)-specific knockout of PDGFR-β was also reported to affect pathological angiogenesis in tumors but did not affect animal survival or normal tissue functions.^31^ However, the impact of endothelial PDGFR-β on the regulation of subchondral H-type vessel biology during OA development remains unclear.

Talin, a 270 kD dimeric adaptor protein, is composed of a C-terminal flexible rod field and an N-terminal globular head domain that can be detached by calpain 2 cleavage.^32^ The N-terminal head of talin harbors an analogous FERM domain that comprises several binding sites for focal adhesion kinase (FAK), actin, layilin, integrin β3, etc.^33^ Talin1 in endothelial cells is crucial for postnatal and embryonic angiogenesis.^34,35^ In the current study, we revealed that subchondral PDGFR-β expression is increased in OA patients, mice with post-traumatic OA, and aged mice. Deletion or specific inhibition of PDGFR-β in ECs attenuated OA in rodents through PDGFR-β/talin1/FAK complex-mediated angiogenesis in subchondral bone. Thus, these results demonstrate the crucial impact of PDGFR-β on OA and provide a potential novel and promising therapeutic target for OA.

## Results

### PDGFR-β in subchondral bone is elevated during osteoarthritis pathogenesis in both humans and mice, with high expression in CD31^hi^Emcn^hi^ ECs

**Fig. 1:**
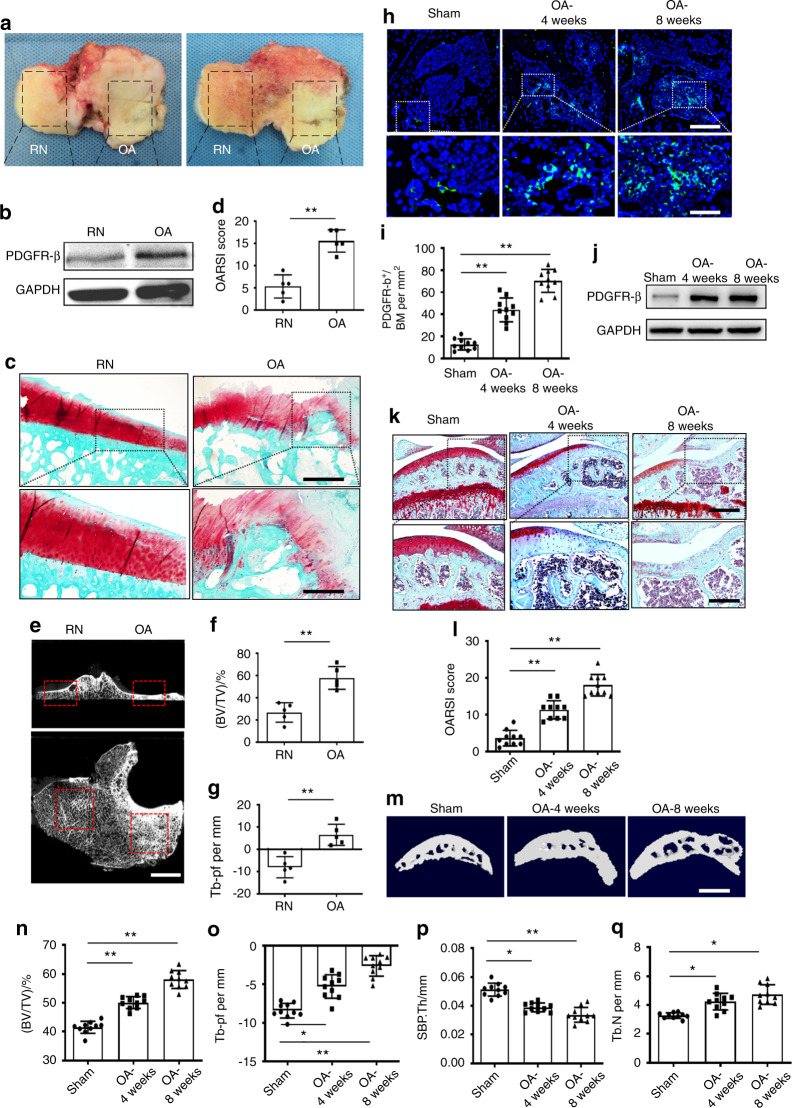
Subchondral PDGFR-β is elevated during OA pathogenesis in humans and mice. **a** Gross inspection of tibial plateau samples acquired from patients who underwent total joint arthroplasty. RN = relatively normal part; OA = damaged osteoarthritic part. **b** Western blot examination of subchondral PDGFR-β expression in OA or RN tissues. **c** Safranin O and fast green (SOFG) staining of subchondral bone in the OA and RN parts from human osteoarthritic tibial plateau samples (upper) with the magnified area (bottom) in the boxed area in the upper image; proteoglycan (red) and bone (green). Scale bar, bottom 50 µm; top 100 µm. **d** OARSI-modified Mankin scores of articular cartilage. *n* = 5. **e** μCT images of subchondral bone from human osteoarthritic samples. Upper: coronal μCT images of subchondral bone; bottom: cross-section of μCT images of subchondral bone; RN part (left box area) and OA part (right box area). Scale bar, 500 μm. Quantitative analysis of BV/TV (**f**) and Tb.pf (**g**). **h** Immunostaining of subchondral PDGFR-β-positive cells (upper) in mice with a magnified area (bottom) in the boxed area in the upper image in the sham and 4-week and 8-week post-ACLT groups. Scale bar, bottom 50 µm; top 100 µm. **i** Quantification of subchondral PDGFR-β-positive cells in the mice in the sham and 4-week and 8-week post-ACLT groups. *n* = 10. **j** Western blot examination of subchondral PDGFR-β expression in the mice in the sham and 4-week and 8-week post-ACLT groups. **k** SOFG staining (upper) with the magnified area (bottom) in the boxed area in the upper image; proteoglycan (red) and bone (green). Scale bar, bottom 50 µm; top 100 µm. **l** OARSI-modified Mankin scores of articular cartilage from the mice in the sham and 4-week and 8-week post-ACLT groups. *n* = 10. μCT images of medial subchondral bone from the sham and 4-week and 8-week post-ACLT groups (**m**) and quantitative analysis of BV/TV (**n**), Tb.pf (**o**), SBP.Th (**p**), and Tb.N (**q**). Scale bar, top 500 µm. *n* = 10. Sham = sham controls. *P < 0.05 and **P < 0.01 compared to the sham group or as denoted by bars

We first detected alterations in PDGFR-β levels in the subchondral bone of humans with OA. Western blot results showed that the PDGFR-β level was remarkably elevated in the osteoarthritic part of the human subchondral bone compared with the relatively normal (RN) part (Fig. 1a, b).^7^ This elevation of PDGFR-β was accompanied by subchondral bone sclerosis and overlying cartilage degeneration (Fig. 1c–g). We then assessed the expression of PDGFR-β in an anterior cruciate ligament transection (ACLT)-induced mouse model. In parallel with the above findings, PDGFR-β expression was elevated dramatically in the mice with OA relative to the sham controls (Fig. 1h–j). This change was also accompanied by subchondral bone deterioration and articular cartilage degradation (Fig. 1k–q). Furthermore, we observed similar changes in PDGFR-β expression and subchondral bone microarchitecture in older mice compared with the sham controls (Fig. S1a–i). These results demonstrate that elevated PDGFR-β levels are strongly correlated with the deterioration of subchondral bone and thus the subsequent degeneration of overlying cartilage during OA progression.

**Fig. 2:**
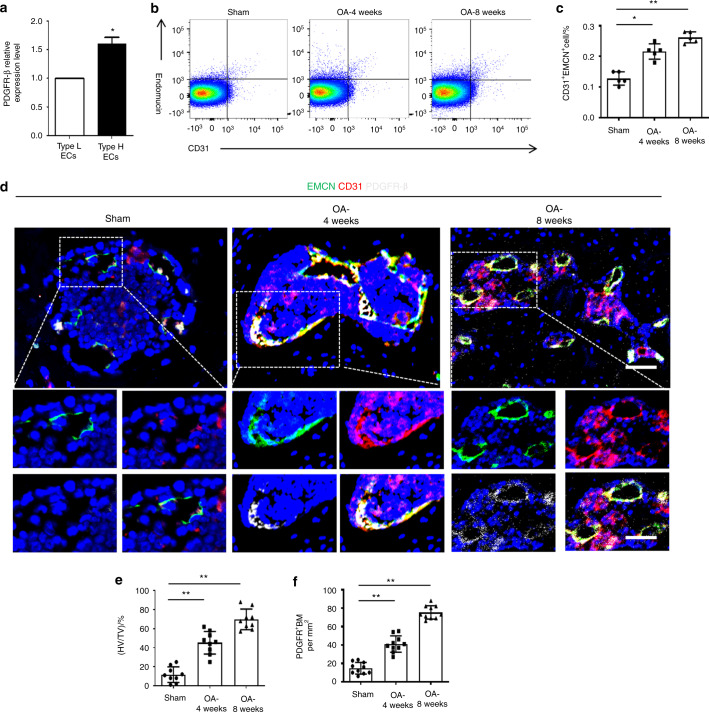
PDGFR-β is primarily expressed in CD31^hi^Emcn^hi^ endothelial cells (ECs). **a** qRT‒PCR examination of PDGFR-β in CD31^hi^Emcn^hi^ ECs and CD31^lo^Emcn^lo^ ECs of bone marrow from normal mice. FACS analysis (**b**) and quantification (**c**) of CD31^hi^Emcn^hi^ ECs isolated from the bone marrow of the sham and 4-week-old and 8-week-old ACLT groups. *n* = 5 (5 mice with 10 subchondral bone specimens). **d** Confocal images (upper) with the magnified area (bottom) in the boxed area in the upper image of H-type vessels (endomucin: green; CD31: red; merge: yellow) and PDGFR-β^+^ cells (white) of the sham and 4-week and 8-week post-ACLT groups. Scale bar, bottom 50 µm; top 100 µm. **e**, **f** Quantification of H-type vessel volume and PDGFR-β^+^ cells in the sham and 4-week and 8-week post-ACLT groups. *n* = 10. Sham = sham controls. TV = total vessel volume; HV = H-type vessel volume. *P < 0.05 and **P < 0.01 compared to the sham group or as denoted by bars

H-type vessels (CD31^hi^Emcn^hi^ vessels) are involved in osteogenesis.^16,17^ Previously, we found that PDGFR-β signaling promotes H-type vessel formation in osteoporosis.^24^ Thus, we explored the correlation between the PDGFR-β level and CD31^hi^Emcn^hi^ endothelial cells (ECs). As previously described,^36^ we identified CD31^hi^Emcn^hi^ and CD31^lo^Emcn^lo^ ECs through fluorescence-activated cell sorting (FACS) and found that PDGFR-β expression in the CD31^hi^Emcn^hi^ ECs was dramatically higher than that in the CD31^lo^Emcn^lo^ ECs, as revealed by qPCR analysis (Fig. 2a). Notably, we found that there were significantly more CD31^hi^Emcn^hi^ ECs in the ACLT-treated mice than in the sham controls (Fig. 2b, c). Triple immunostaining of endomucin, CD31, and PDGFR-β showed that subchondral H-type vessels were highly surrounded with PDGFR-β (Fig. 2d–f). Taken together, these data suggest that PDGFR-β in CD31^hi^Emcn^hi^ ECs may play an essential role in OA pathogenesis.

### Endothelial PDGFR-β deletion prevents the onset of OA in mice

**Fig. 3:**
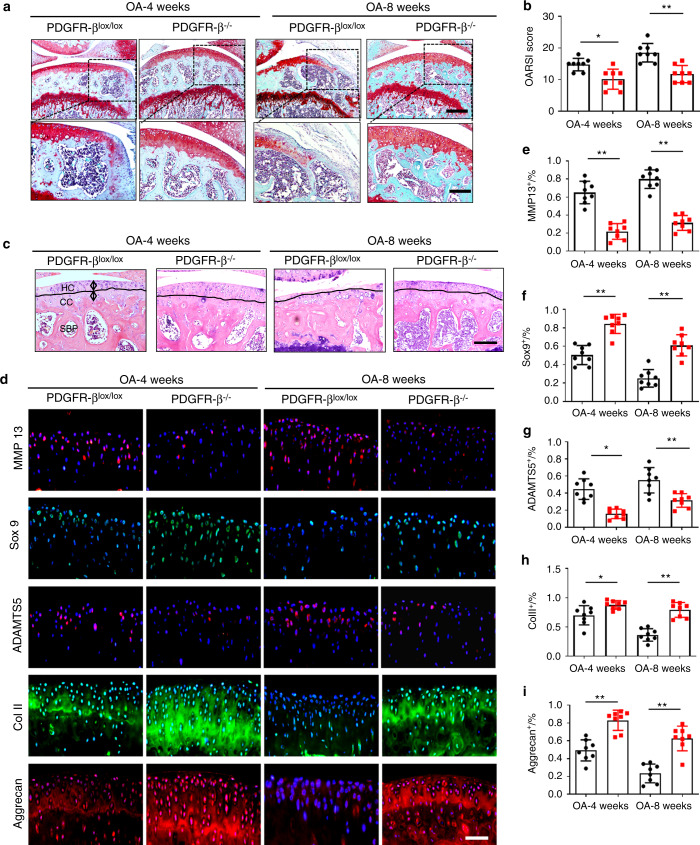
PDGFR-β deletion in ECs prevents the onset of OA. **a** SOFG (upper) with the magnified area (bottom) in the boxed area in the upper image of articular cartilage in the PDGFR-β^lox/lox^ and PDGFR-β^−/−^ mice at 4 weeks and 8 weeks post-ACLT; bone (green) and proteoglycan (red). Scale bar, top 100 µm; bottom 50 µm. **b** OARSI-modified Mankin scores of cartilage in the PDGFR-β^lox/lox^ and PDGFR-β^−/−^ mice at 4 weeks and 8 weeks post-ACLT. *n* = 8. **c** H&E staining where calcified cartilage (CC) and hyaline cartilage (HC) are separated by black dashed lines in the PDGFR-β^lox/lox^ and PDGFR-β^−/−^ mice at 4 weeks and 8 weeks post-ACLT. Scale bars, 50 μm. **d**–**i** Immunofluorescence staining and quantification of MMP-13, Sox9, ADAMTS 5, Col II, and aggrecan in articular cartilage from the PDGFR-β^lox/lox^ and PDGFR-β^−/−^ mice at 4 weeks and 8 weeks post-ACLT. *n* = 8. Scale bar, 50 µm. *P < 0.05 and **P < 0.01 compared to the PDGFR-β^lox/lox^ controls or as denoted by bars

We then examined whether the deletion of PDGFR-β in ECs could ameliorate OA pathogenesis. EC-specific PDGFR-β knockout mice (PDGFR-β^−/−^) were generated by mating loxP-flanked PDGFR-β allele (PDGFR-β^lox/lox^) mice and Cdh5 (PAC)-Cre transgenic mice. The deletion of PDGFR-β in ECs was confirmed in 8-week-old mice using qRT‒PCR (Fig. S2a). It has been demonstrated that PDGFR-β depletion in ECs does not affect basal vessel formation or normal tissue functions.^31^ We also used mice with body weights and limb lengths comparable with those of the littermate controls (Fig. S2b–d). ACLT surgery was performed on 3-month-old PDGFR-β-KO mice and their littermates (controls). We showed that the loss of PDGFR-β in ECs ameliorated OA pathogenesis at 4 and 8 weeks post-operation, as reflected by reduced cartilage degradation and significantly lower Osteoarthritis Research Society International (OARSI) scores (Fig. 3a, b).^37^ Additionally, hematoxylin and eosin (HE) staining showed that PDGFR-β depletion in ECs rescued the upward moving tidemark, that is, the thickening of the calcified cartilage zone in the PDGFR-β^lox/lox^ littermates (Fig. 3c). Moreover, we observed that the increased levels of MMP13 (matrix metallopeptidase 13) and ADAMTS5 (a disintegrin and metalloproteinase with thrombospondin motifs 5) and the decreased levels of SOX 9 (SRY-box transcription factor 9), aggrecan and collagen II in the PDGFR-β^lox/lox^ controls were normalized by PDGFR-β deficiency in ECs (Fig. 3d–i). We also observed alleviated cartilage degeneration in older PDGFR-β^−/−^ mice compared with their littermate controls (Fig. S3a–c). These data indicate that OA progression is diminished in the mutant mice relative to the controls.

### PDGFR-β depletion in ECs reduces angiogenesis and osteogenesis in the subchondral bone of mice with OA

**Fig. 4:**
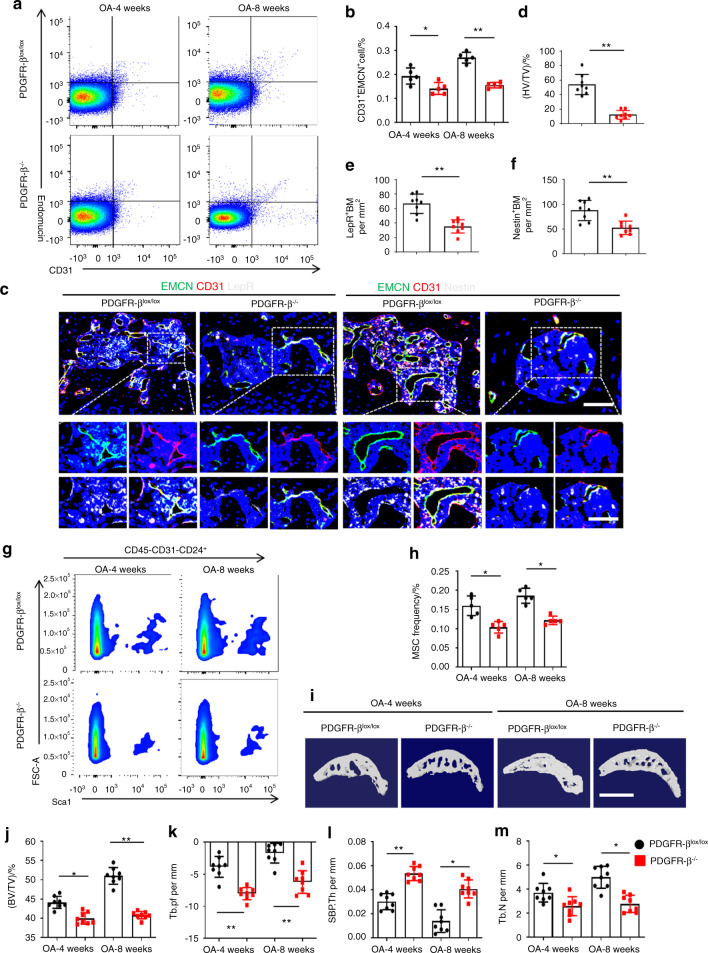
Endothelial PDGFR-β regulates subchondral H-type vessels and the linked MSCs. FACS analysis (**a**) and quantification (**b**) of subchondral CD31^hi^Emcn^hi^ ECs isolated from the PDGFR-β^lox/lox^ and PDGFR-β^−/−^ mice at 4 weeks and 8 weeks post-ACLT. *n* = 5 (5 mice with 10 subchondral bone specimens). **c** Confocal images (upper) with the magnified area (bottom) in the boxed area in the upper image of H-type vessels (merge: yellow; endomucin: green; CD31: red) and LepR^+^ cells (left part, white) and Nestin^+^ cells (right part, white) in the subchondral bone from the PDGFR-β^lox/lox^ and PDGFR-β^−/−^ mice at 4 weeks post-ACLT. Scale bar, bottom 50 µm; top 100 µm. Quantification of subchondral H-type vessel volume (**d**), LepR^+^ cells (**e**) and Nestin^+^ cells (**f**) from the PDGFR-β^lox/lox^ and PDGFR-β^−/−^ mice at 4 weeks post-ACLT. *n* = 8. FACS (**g**) and quantification (**h**) of subchondral MSCs (CD45^−^CD31^−^Sca1^+^CD24^+^) isolated from the PDGFR-β^lox/lox^ and PDGFR-β^−/−^ mice at 4 weeks and 8 weeks post-ACLT. μCT images of medial subchondral bone (**i**) and quantitative analysis of BV/TV (**j**), Tb.pf (**k**), SBP.Th (**l**), and Tb.N (**m**) in the subchondral bone from the PDGFR-β^lox/lox^ and PDGFR-β^−/−^ mice at 4 weeks and 8 weeks post-ACLT. *n* = 8. Scale bar, 500 µm. *P < 0.05 and **P < 0.01 compared to the PDGFR-β^lox/lox^ controls or as denoted by bars

Previous studies have demonstrated that the deterioration of subchondral bone possibly precedes the degeneration of overlying cartilage.^38,39^ Subchondral bone deterioration initiates a cascade of events contributing to cartilage degradation and OA pathogenesis.^4,6,19^ Here, we further investigated whether the loss of function of PDGFR-β in ECs affects the formation of subchondral H-type vessels and MSCs in ACLT-induced mice. FACS was used to sort subchondral CD31^hi^Emcn^hi^ ECs from the PDGFR-β^−/−^ mice and the PDGFR-β^lox/lox^ controls. The results showed that the number of CD31^hi^Emcn^hi^ ECs in the PDGFR-β^−/−^ mice was markedly decreased relative to that in the PDGFR-β^lox/lox^ littermate controls at both 4 and 8 weeks after surgery (Fig. 4a, b). We also observed that subchondral PDGFR-β was elevated in the PDGFR-β^−/−^ mice after ACLT, whereas no difference was observed between the preoperation and 4 weeks postoperation groups (Fig. S4a, b). This finding demonstrated that the elevation of PDGFR-β in subchondral bone might also be derived from other nonendothelial sources, such as pericytes;^29^ however, endothelial PDGFR-β in CD31^hi^Emcn^hi^ ECs was the primary modulator of H-type vessel formation. Triple immunostaining of CD31, endomucin, and leptin receptor (LepR) or Nestin demonstrated that the amount of subchondral CD31^hi^Emcn^hi^ vessels, as well as the accompanying LepR- or Nestin-positive MSCs, was dramatically reduced in the PDGFR-β^−/−^ ACLT-induced mice compared with their PDGFR-β^lox/lox^ ACLT littermates (Fig. 4c–f and Fig. S4c–f). Flow cytometry analysis also revealed a significant reduction in the number of MSCs (CD45^−^CD31^−^Sca1^+^CD24^+^) in the bone marrow of the PDGFR-β^−/−^ mice relative to the littermate controls after ACLT (Fig. 4g, h).^40,41^ Consistently, a reduction in subchondral MSCs and H-type vessels was also observed in the older PDGFR-β^−/−^ mice compared with the PDGFR-β^lox/lox^ controls (Fig. S5a–d). Furthermore, the depletion of PDGFR-β in ECs reversed uncoupled subchondral bone remodeling in the PDGFR-β^lox/lox^ controls, as demonstrated by the normalization of trabecular pattern factor (Tb. Pf), subchondral bone plate thickness (SBP.Th), trabecular bone number (Tb.N), and bone volume/total tissue volume (BV/TV) (Fig. 4i–m). Of interest, the results of gait analysis and the von Frey test showed that PDGFR-β deletion in ECs could alleviate joint pain during OA development (Fig. S6a–e and Fig. S7a, b). These results demonstrate that endothelial PDGFR-β regulates subchondral angiogenesis coupled with osteogenesis during OA pathogenesis.

### Endothelial PDGFR-β promotes angiogenesis through the formation of the PDGFR-β/talin1/FAK complex

**Fig. 5:**
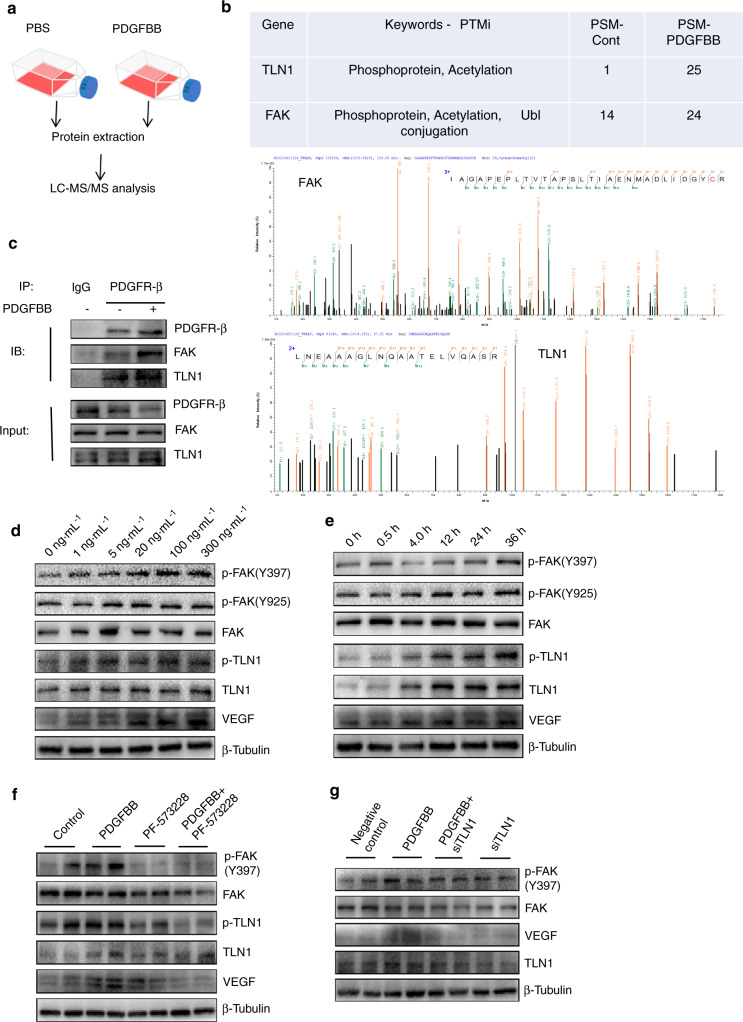
PDGFR-β interacts with FAK and TLN1 in ECs. **a** Strategy for identifying PDGFR-β binding proteins using LC-MS/MS analysis. Bone morrow endothelial cells (BMECs) treated with PDGF-BB (300 ng·mL^−1^) for 24 h were subjected to immunoprecipitation (IP) with an antibody against PDGFR-β followed by LC-MS/MS analysis. **b** The PDGFR-β binding proteins FAK and TLN1 were identified with LC-MS/MS analysis. **c** PDGFR-β was immunoprecipitated from BMECs with an anti-PDGFR-β antibody. The presence of FAK, TLN1, and PDGFR-β in these immunoprecipitates was evaluated with immunoblotting. Western blot analysis of FAK, TLN1, and VEGF in the BMECs treated with different PDGF-BB doses (0-300 ng·mL^−1^) (**d**) or for different times at 0 h–36 h (**e**). Western blot analysis of FAK, TLN1, and VEGF in the BMECs treated with FAK inhibitors (**f**) or TLN1 siRNA (**g**)

To explore the underlying mechanism by which endothelial PDGFR-β regulates angiogenesis, we subjected bone morrow endothelial cells (BMECs) treated with PDGF-BB (300 ng·mL^−1^) to immunoprecipitation (IP) with an antibody against PDGFR-β followed by liquid chromatography with tandem mass spectrometry (LC‒MS/MS) analysis to detect PDGFR-β-interacting proteins in BMECs. Table 1, 2. A total of 1553 PDGFR-β-binding proteins were identified in the BMECs with or without PDGF-BB treatment (Table S1). Among these candidates, talin1 showed the largest change (25-fold increase) with PDGF-BB treatment compared to PBS treatment. FAK is considered a key partner for talin1-regulated vessel formation.^42^ A 2-fold increase in FAK was also observed in the BMECs treated with PDGF-BB. Thus, talin1 and FAK were selected for further study (Fig. 5a, b). The binding of PDGFR-β, talin1, and FAK was confirmed through IP assays, as demonstrated by the purified complex of PDGFR-β/talin1/FAK, suggesting that talin1 and FAK are potential binding partners of PDGFR-β (Fig. 5c). As expected, the binding of PDGFR-β, talin1 and FAK was stronger with PDGF-BB treatment. Next, we tested whether the PDGFR-β/talin1/FAK complex was activated by PDGF-BB. Western blot assays revealed that phosphorylation of talin1 and FAK was induced by endothelial PDGF-BB/PDGFR-β in a time- and dose-dependent manner (Fig. 5d, e). Furthermore, the activation of the PDGFR-β/talin1/FAK complex promoted VEGF expression in BMECs (Fig. 5e). Both the FAK inhibitor and siRNA-TLN1 abrogated endothelial PDGF-BB/PDGFR-β-induced VEGF expression (Fig. 5f, g).

**Table 1:** The primer sequences selected for qRT‒PCR

PDGFR-β forward	ACATGCCAGGTACTAGGTATGATG
PDGFR-β reverse	ACCCATCTCTCAAAAGCTTATCCC
GAPDH forward	TGTCGTGGAGTCTACTGGTG
GAPDH reverse	GCATTGCTGACAATCTTGAG

**Table 2:** The target sequences used for PDGFR-β knockdown lentivirus or siTLN1

Pdgfrb-RNAi-7	caGGTGGTGTTTGAGGCTTAT
Pdgfrb-RNAi-8	gaGCATCACCATCAGGTGCAT
Pdgfrb-RNAi-9	gaGGAAACCACGCTATGAGAT
TLN1 siRNAs-1 forward	CCUCCAAAGCUUGGCGUAATT
TLN1 siRNAs-1 reverse	UUACGCCAAGCUUUGGAGGTT
TLN1 siRNAs-2 forward	CCAAAUGGCCCAGUACUUUTT
TLN1 siRNAs-2 reverse	AAAGUACUGGGCCAUUUGGTT
TLN1 siRNAs-3 forward	CCAGCUGACCAGUGACUAUTT
TLN1 siRNAs-3 reverse	AUAGUCACUGGUCAGCUGGTT

**Fig. 6:**
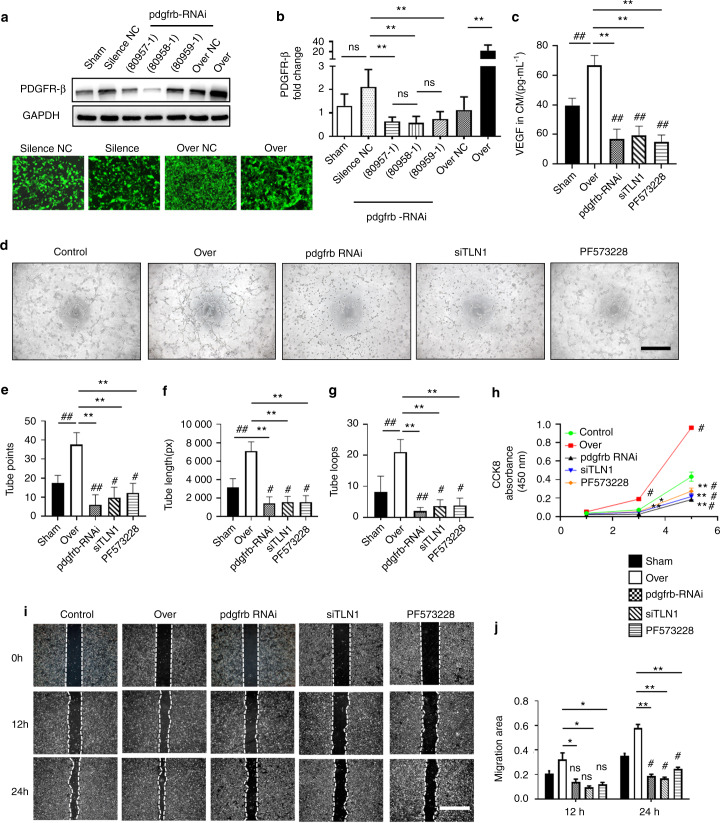
Endothelial PDGFR-β promotes angiogenesis through the PDGFR-β/Talin1/FAK pathway. The efficiency of lentivirus transfection was verified using Western blot analysis (**a**) and qRT‒PCR (**b**). For pdgfrb silencing, 80958-1 exhibited optimum efficiency and was selected for the following experiments. **c** ELISA examination of VEGF expression in the CM of BMECs under different conditions. **d**–**g** Representative images and quantification analysis of tube formation in BMECs. Scale bar, 500 µm. **h** CCK-8 measurement of BMEC proliferation under different conditions. **i**, **j** Scratch wound assay showing BMEC motility under different conditions. Scale bar, 200 μm. Control = CM of BMECs from the normal control group; Over = CM of BMECs transfected with Lv-PDGFR-β; pdgfrb silencing = CM of BMECs transfected with Lv-si PDGFR-β; siTLN1 = CM of BMECs transfected with TLN1 siRNA; PF573228 = CM of BMECs treated with PF573228 (FAK inhibitor). *P < 0.05 and **P < 0.01 compared to the over group. ^#^*P* < .05 and ^##^*P* < .01 compared to the control group

To further confirm the role of the PDGFR-β/talin1/FAK complex in angiogenesis, we overexpressed or silenced PDGFR-β in BMECs by transfecting lentivirus carrying the PDGFR-β gene (Lv-PDGFR-β) or PDGFR-β-specific siRNA (Lv-si PDGFR-β) (Fig. 6a, b). ELISA results confirmed the effect of the PDGFR-β/talin1/FAK complex on VEGF expression in BMECs (Fig. 6c). A tube formation assay with Matrigel revealed that overexpression of PDGFR-β in BMECs resulted in substantial elevation of total loops, total branching points, and total length, whereas interruption of PDGFR-β signaling, such as by loss-of-function PDGFR-β or talin1, and use of a FAK inhibitor decreased these parameters relative to the controls (Fig. 6d–g). The CCK-8 assay showed that the proliferative ability of BMECs was enhanced with PDGFR-β overexpression; however, silencing PDGFR-β or talin1 and the FAK inhibitor impaired the BMEC proliferative ability compared with that of the controls (Fig. 6h). A scratch wound healing assay (Fig. 6i, j) revealed similar results, in which we observed that the motility of BMECs augmented by overexpression of PDGFR-β was blocked by related signaling disruption. Taken together, these data indicate that endothelial PDGFR-β modulates angiogenesis through the PDGFR-β/talin1/FAK pathway.

### Endothelium-specific inhibition of PDGFR-β attenuates OA pathogenesis

**Fig. 7:**
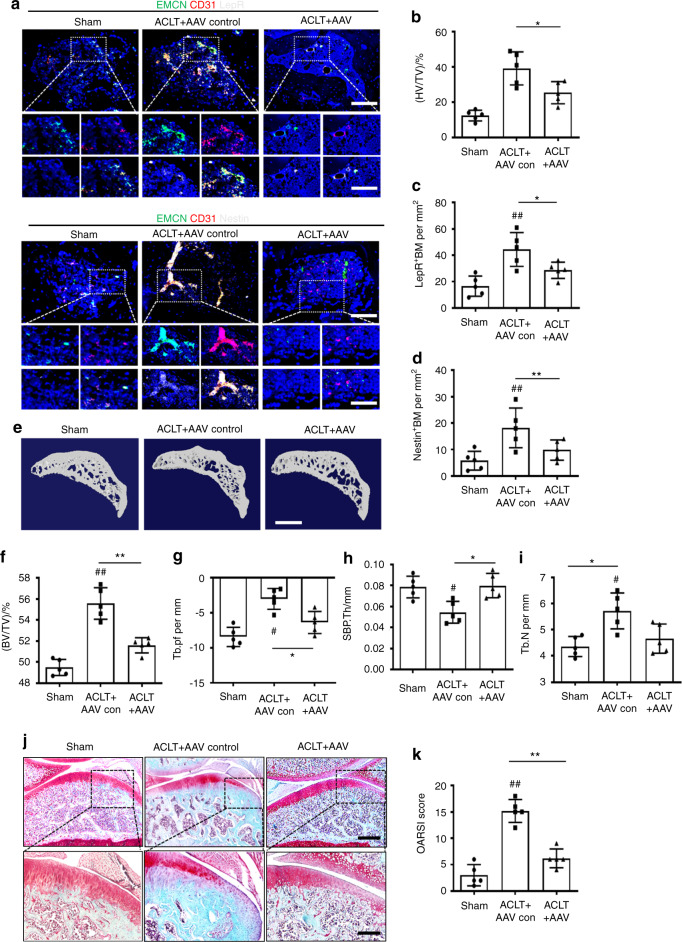
Specific inhibition of PDGFR-β in ECs attenuates OA development. **a** Confocal images (upper) with the magnified area (bottom) in the boxed area in the upper image of H-type vessels (merge: yellow; endomucin: green; CD31: red) and LepR^+^ cells (left part, white) and Nestin^+^ cells (right part, white) in the subchondral bone from the sham, ACLT + AAV control and ACLT + AAV rats at 8 weeks post-ACLT. Scale bar, bottom 50 µm; top 100 µm. Quantification of subchondral H-type vessel volume (**b**) and LepR^+^ cells (**c**) and Nestin^+^ cells (**d**) from the sham, ACLT + AAV control and ACLT + AAV rats at 8 weeks post-ACLT. *n* = 5. μCT images of medial subchondral bone (**e**) and quantitative analysis of BV/TV (**f**), Tb.pf (**g**), SBP.Th (**h**), and Tb.N (**i**) from the sham, ACLT + AAV control and ACLT + AAV rats at 8 weeks post-ACLT. *n* = 5. Scale bar, 500 µm. (**j**) SOFG (upper) with the magnified area (bottom) in the boxed area in the upper image of articular cartilage from the sham, ACLT + AAV control, and ACLT + AAV rats at 8 weeks post-ACLT; proteoglycan (red) and bone (green). Scale bar, bottom 50 µm; top 100 µm. **k** OARSI-modified Mankin scores of articular cartilage in sham, ACLT + AAV control, and ACLT + AAV rats at 8 weeks post-ACLT. *n* = 5. Sham = sham controls; ACLT + AAV control = AAV control-treated ACLT rats; ACLT + AAV = AAV for silencing endothelial PDGFR-β-treated ACLT rats; *P < 0.05 and **P < 0.01 compared to the ACLT + AAV control group. ^#^*P* < 0.05 and ^##^*P* < 0.01 compared to the sham group

To confirm the role of endothelial PDGFR-β in OA, we injected adeno-associated virus (AAV) serotype 9 to silence endothelial PDGFR-β in the subchondral bone of the ACLT-induced rats.^43,44^ Two months after injection, subchondral AAV expression could still be detected (Fig. S8a–c), suggesting that AAV9 administered by local injection in subchondral bone has a long-term effect on subchondral gene expression. We next conducted phenotypic analysis to explore how silencing endothelial PDGFR-β in subchondral bone affects OA pathogenesis. Triple immunostaining of endomucin, CD31, and LepR or Nestin showed that inhibition of PDGFR-β in ECs normalized the number of subchondral CD31^hi^Emcn^hi^ vessels and LepR- or Nestin-positive MSCs relative to those of the vehicle-treated ACLT rats (Fig. 7a–d). Furthermore, endothelial-specific inhibition of PDGFR-β reversed uncoupled subchondral bone remodeling in the vehicle-treated ACLT rats, as demonstrated by the normalization of trabecular pattern factor (Tb.Pf), subchondral bone plate thickness (SBP.Th), bone volume/total tissue volume (BV/TV), and trabecular bone number (Tb.N) (Fig. 7e–i). More importantly, inhibition of endothelial PDGFR-β alleviated the onset of OA; suppression of PDGFR-β in ECs led to a decrease in cartilage degradation and OARSI scores (Fig. 7j, k). Cartilage marker staining showed that the decreased aggrecan, collagen II, and SOX 9 levels and elevated MMP13 and ADAMTS5 levels in the vehicle-treated ACLT rats returned to normal after treatment with AAV9 (Fig. S9a–f). Together, our exploration of endothelial inhibition of PDGFR-β in vivo demonstrates that PDGFR-β could serve as an effective target for OA treatment.

## Discussion

No effective disease-modifying drugs are currently available for the treatment of OA. Although cartilage degradation is the crucial characteristic of OA, it is now considered that the whole joint, including subchondral bone, is involved in OA pathogenesis.^3–5,45^ Throughout life, subchondral bone cells and joint chondrocytes sense and react accordingly to acute or chronic mechanical stimulation. OA develops if reparative or homeostatic processes cannot sufficiently compensate for the deteriorative mechanisms.^46^ In this process, it is important to determine the driving factor that leads to progressive deterioration of the cartilage, as cartilage scarification does not inevitably result in OA development.^47^ Thus, the most effective therapy for OA should alleviate the driving factor rather than just protect articular cartilage. Regarding subchondral bone, recent observations have demonstrated that OA is involved in early bone loss resulting from enhanced bone remodeling, followed by decreased bone turnover contributing to sclerosis of the subchondral bone.^5,6^ Thus, a therapeutic strategy might involve the following two aspects: inhibition of early excessive bone resorption and late aberrant bone formation. Of note, inhibition of elevated bone resorption using bisphosphonate (BP) in knee OA patients can attenuate the progression of the disease and further decrease the risk of knee arthroplasty.^39,48^ Due to the current incurability and repeated relapses of OA, we believe there are no absolute early or late stages of OA, which should be present in a mixed format in various stages or in different regions of subchondral bone. Further study of the effect of interrupting PDGFR-β-linked bone formation on OA in the clinic would be promising. In the current study, we found that PDGFR-β expression was remarkably elevated in human osteoarthritic subchondral bone and subchondral bone from aged mice and those with post-traumatic OA. Depletion and specific inhibition of PDGFR-β in ECs attenuated OA in rodents.

Endothelial PDGFR-β enhanced H-type vessel-dependent abnormal subchondral bone formation in rodents with post-traumatic or age-dependent OA. Abnormal subchondral bone formation has a triggering role in OA development,^1,3,6^ which alters the mechanical loading on the overlying cartilage, thus inducing the degradation of overlying cartilage. Osteogenesis, both normal and abnormal, is often linked with angiogenesis.^12,15^ A novel vessel subtype named H-type vessels has recently been shown to combine angiogenesis and osteogenesis.^16,17^ We have recently revealed that an increased amount of subchondral H-type vessels forms a positive feedback loop with MSCs during OA development and that PDGF-BB/PDGFR-β signaling is essential for H-type vessel enhancement in osteoporosis.^18,19,24^ Moreover, a recent study by Su W et al. revealed that PDGF-BB derived from mononuclear preosteoclasts was a crucial driver of pathological angiogenesis-dependent subchondral bone formation during OA development.^29^ These researchers found that specific deletion of PDGF-BB in preosteoclasts alleviated subchondral bone deterioration and articular cartilage degeneration, whereas enhancement of PDGF-BB expression in preosteoclasts in transgenic mice was sufficient to spontaneously induce OA. However, the effect of subchondral PDGFR-β on subchondral H-type vessels and OA pathogenesis is still unclear. Previous studies have demonstrated that PDGFR-β is expressed in ECs and that EC-specific knockout of PDGFR-β affects pathological angiogenesis in tumors but does not affect animal survival or normal tissue functions.^31,49^ Here, we found that PDGFR-β in subchondral bone was predominantly expressed in CD31^hi^Emcn^hi^ ECs and that PDGFR-β was markedly increased in the subchondral bones of humans with OA, as well as in older mice and mice with trauma-induced OA. Notably, depletion of PDGFR-β in ECs or endothelium-specific inhibition of PDGFR-β by local injection of AAV9 in subchondral bone effectively attenuated aberrant H-type vessel-dependent subchondral bone formation and OA pathogenesis. We also found that subchondral PDGFR-β was increased in the PDGFR-β^−/−^ mice after ACLT, whereas no difference was observed in the groups preoperatively or 4 weeks postoperatively. This finding demonstrated that the increased PDGFR-β in subchondral bone might also be derived from nonendothelial sources, such as pericytes.^29^ PDGFR-β is extensively expressed on the surface of pericytes and is required for the recruitment of pericytes to the sprouting angiogenic front.^50^ Thus, we believe that during OA development, excessive PDGF-BB derived from subchondral mononuclear preosteoclasts stimulates PDGFR-β in CD31^hi^Emcn^hi^ ECs to promote the proliferation of CD31^hi^Emcn^hi^ ECs and activates PDGFR-β in pericytes to initiate the mobilization and recruitment of pericytes to sprouting CD31^hi^Emcn^hi^ ECs, which synergistically enhances abnormal H-type vessel-dependent subchondral bone formation in OA. In addition, increased attention has been paid to the development of new drugs to alleviate OA-associated pain. Indeed, joint pain is the defining symptom of OA, but its origin remains obscure. Articular cartilage has no nerve or blood vessel innervation under normal situations. It is also believed that OA is a noninflammatory form of arthritis, with at least no synovitis at the early stage. In the clinic, it is also believed that no synovitis exists in patients with OA of grade 1 according to the Kellgren-Lawrence (KL) classification, although 38% of patients with OA of grades 2–3 have infrapatellar synovitis.^51^ Interestingly, an increasing number of studies have shown that subchondral bone deterioration may be associated with the origin of pain at the early stage of OA.^3,52,53^ Thus, interventions enabling the restabilization of subchondral bone homeostasis might be effective for pain relief during OA development. In the current study, the results of gait analysis and the von Frey test showed that PDGFR-β deletion in ECs attenuated OA through the reversal of angiogenesis-dependent subchondral bone deterioration and alleviated pain during the onset of OA. These results suggest that subchondral endothelial PDGF-BB/PDGFR-β can modulate OA pathogenesis through the regulation of H-type vessel-dependent aberrant subchondral bone formation. However, how endothelial PDGFR-β mediates the formation of subchondral H-type vessels remains unclear.

Endothelial talin1 is crucial for postnatal and embryonic angiogenesis.^34,35^ Two talin isoforms exist in vertebrates: talin1 and talin2. Talin2 is 86% similar and 74% identical to talin1. Talin2 can compensate for and rescue the phenotype of talin1 loss in cells.^54–56^ It has been reported that talin1 but not talin2 is expressed in all cell types.^55,57,58^ Interestingly, ECs do not express talin2, which indicates that ECs lacking talin1 will lose the function of the cell mobilization and recruitment required for angiogenesis.^34^ Talin1 is necessary for the maintenance of cell migration, cell spreading, and focal adhesion (FA) formation.^54,56^ A previous study demonstrated that during the promotion of angiogenesis, talin1 can directly interact with FAK, supporting the critical impact of the FAK-talin interaction on blood vessel formation.^59^ In this study, we found that endothelial PDGFR-β promotes angiogenesis through the formation of the PDGFR-β/talin1/FAK complex, which enhances VEGF expression in BMECs. Notably, silencing of PDGFR-β or talin1 and a FAK inhibitor abrogated endothelial PDGFR-β/talin1/FAK-induced angiogenesis. However, we cannot exclude the possibility that there are proteins other than talin1 and FAK among the 1553 proteins identified to bind to PDGFR-β by LC‒MS/MS analysis that mediate the effects of PDGF-BB/PDGFR-β on angiogenesis, which is worthy of future study.

Additionally, the sensory nerve regulation of bone formation has recently been intensely investigated. Sensory nerves can sense a bone-forming “signal”, that is, prostaglandin E2 (PGE2), and PGE2 then binds with its EP4 receptor to activate the phosphorylation of CREB1 in the hypothalamus, thus maintaining bone mass by mediating sympathetic nerve activity.^60^ Furthermore, sensory nerve EP4/PGE2 signaling can determine MSC osteogenic commitment.^40^ Some promising molecules, such as nerve growth factor (NGF) and netrin 1, have been proven to trigger subchondral sensory nerve innervation during OA development.^53,61^ Further study focusing on the role of the sensory nerve in OA and its potential mechanism would help elucidate the OA pathomechanisms and the development of new disease-modifying drugs for OA.

To conclude, we identified endothelial PDGFR-β as a novel therapeutic target for OA. PDGFR-β in ECs promotes subchondral H-type vessel formation and aberrant bone formation through the PDGFR-β/talin1/FAK pathway. Loss of function and specific inhibition of PDGFR-β in ECs attenuated OA pathogenesis. Taken together, our study provides mechanistic insight into how endothelial PDGFR-β modulates vessel formation during coupling with bone formation in OA development. PDGFR-β is therefore a potential effective target for OA treatment.

## Materials and Methods

### Mice, rats, and clinical samples

LoxP-flanked PDGFR-β and Cdh5-Cre were obtained from Cyagen (Stock No. 017986, China). First, mice carrying loxP-flanked PDGFR-β alleles (PDGFR-β^lox/lox^) and Cdh5-Cre transgenic mice were crossed to obtain Cdh5-Cre::PDGFR-β^lox/−^ mice, which were then mated with PDGFR-β^lox/lox^ mice to obtain Cdh5-Cre:PDGFR-β^lox/lox^ mice (PDGFR-β^−/−^). For the endothelium-specific PDGFR-β knockout experiment, 3-month-old male PDGFR-β^lox/lox^ mice and PDGFR-β^−/−^mice were used to perform anterior cruciate ligament transection surgery (ACLT) to observe post-traumatic OA in each independent experiment (*n* = 8 per group). Two-month-old and 15-month-old male PDGFR-β^lox/lox^ mice and PDGFR-β^−/−^mice were used to observe age-related OA for each independent experiment (*n* = 8 per group). PCR analyses of genomic DNA were used to determine the genotypes of the mice using the following primers:

**Table 3:** The primer sequences used to determine the genotypes of the mice

Gene name	Sequence
pdgfrb forward	ACATGCCAGGTACTAGGTATGATG
pdgfrb reverse	ACCCATCTCTCAAAAGCTTATCCC
Cdh5 forward	CCAGGCTGACCAAGCTGAG
Cdh5 reverse	CCTGGCGATCCCTGAACA

All 1-month-old (*n* = 10), 3-month-old (*n* = 10), 6-month-old (*n* = 10), and 15-month-old (*n* = 10) male C57BL/6J (WT) mice and 3-month-old male SD (Sprague Dawley) rats (*n* = 5 in each group) were purchased from the animal center of Southern Medical University, Guangzhou, China.

ACLT was introduced to establish a post-traumatic OA model as previously described.^6,18,62^ In the OA group, ACLT surgery was carried out on the left knee joint under the supervision of a surgical loupe. For rodents in the sham group, the joint capsule was opened and then sutured in the left knee. After surgery, the rodents were randomized to plastic cages based on body weight and the study plan, allowing them to move around freely in cages. The mice were euthanized at 4 or 8 weeks after surgery, and the rats were sacrificed at 8 weeks post-operation. The experimental protocols were approved by the Institutional Animal Care and Use Committee of Southern Medical University.

Human specimens were collected from OA patients who underwent total knee arthroplasty (TKA). The patients were aged 65–75 years with symptomatic radiographic tibial knee OA and no other systemic diseases (*n* = 5, 3 female and 2 male). The medial compartment of specimens with severe cartilage injury was used for the OA group, while the lateral compartment of specimens with intact cartilage coverage was used for the RN group. CT scanning and histological examination were performed on the specimens. The personal information was anonymized. This study was approved by the ethical medical committee of Nanfang Hospital.

### Local injection of AAV in subchondral bone

Recombinant AAV for silencing endothelial PDGFR-β was locally injected into subchondral bone in vivo. The pdgfrb shRNA virus (AAV2/9-TIE1p-EGFP-miR30shRNA(pdgfrb)) and the controls (AAV2/9-TIE1p-EGFP) were constructed by Obio Technology (China). The target sequence of pdgfrb shRNA was CAGGTGGTGTTTGAGGCTTAT, and the control was AAV2/9-TIE1p-EGFP. The pdgfrb shRNA sequence was inserted into the intron of the TIE1p promoter to drive the expression of EGFP, which was utilized to detect AAV-induced pdgfrb shRNA expression. ACLT surgery was carried out on the left knee of 3-month SD rats to induce OA. Ten days later, 5 × 10^9^ AAV particles in a 30 µl volume were injected into the subchondral bone of the left ACLT-treated knee in the SD rats.

### Histochemical, immunofluorescence, and histomorphometric analyses

The rodents were anesthetized, and then, the knees were harvested after heart perfusion, fixed for 24 h in 4% paraformaldehyde, and decalcified for 3 weeks in 10% EDTA (pH 7.4). The samples were fixed in OCT (optimal cutting temperature) compound (Sakura Finetek) or paraffin. Then, the medial compartment of the samples was longitudinally oriented and cut to 4 μm. Hematoxylin and eosin (H&E) staining was performed to calculate the ratio of the thickness of calcified cartilage (CC) and hyaline cartilage (HC) in cartilage. The tidemark line shows the separation of CC and CC by H&E staining. Safranin O/Fast Green (SOFG) staining was performed to examine proteoglycans in cartilage. The tissue samples were mounted after being dehydrated (ethanol). Images were acquired by microscopy (Olympus). OARSI (Osteoarthritis Research Society International-modified Mankin criteria) scores were measured as previously described. Immunostaining staining was performed using the following primary antibodies: PDGFR-β (Santa Cruz, 1:50, sc-374573), endomucin (Santa Cruz, 1:50, sc-65495), CD31 (Abcam, 1:200, ab222783), nestin (Santa Cruz, 1:50, sc-58813), lepR (Santa Cruz, 1:50, sc-8391), MMP13 (Santa Cruz, 1:50, sc-30073), ATAMDTS (Santa Cruz, 1:200, #C04789), SOX9 (Santa Cruz, 1:50, sc-166505), Aggrecan (Santa Cruz, 1:50, sc-166951), and COL II (Santa Cruz, 1:50, sc-52658) at 4 °C overnight. Secondary antibodies conjugated with fluorescence tags were incubated at room temperature (RT) for 1 h in the dark. Nuclei were labeled with DAPI (DAPI; Servicebio) before imaging. The entire subchondral bone area of the slices was then microphotographed for histomorphometric measurements. Olympus confocal microscopy was employed to calculate positive subchondral cells. The number of entire subchondral positive cells was counted for each specimen, and five sequential samples were measured per rodent in each group. Quantitative analysis was performed in a blinded manner using ImageJ software.

### Microcomputed tomography analysis

After soft tissue dissection, the harvested knee joints were fixed in 70% ethanol overnight. We then scanned and reconstructed the samples with high-resolution microcomputed tomography (CT) (SkyScan 1172) and CT reconstruction software (NRecon v1.6). Then, CTAn v1.9 and μCTVol v2.0 were used for three-dimensional model visualization and further data analysis. The parameters of the scanner were as follows: 50 kVp voltage, 200 μA current, and 9 μm per pixel resolution. We selected the region of interest as the entire subchondral bone of the specimens. Tb.Pf, BV/TV, Tb.N, and SBP.Th were measured.

### Gait analysis

We used a highly sensitive, automated computer-assisted method to determine the impact of endothelium-specific PDGFR-β knockout on gait coordination according to a previously described protocol^63^ (Rodent Gait Behavior Analyzer (GAT-RGBA); Shenzhen Giant (Ju’An) Technologies Co., Ltd.). The CatWalk system is a complex device that quantitatively measures footfall and motor performance. First, we trained animals to willingly go across the illuminated glass platform. Next, a high-speed high-resolution camera captured the animal paw location when the animals walked on the illuminated glass walkway. For a representative number of completed runs per animal, up to 20 runs were recorded for each animal. Next, gait analysis was performed. Paw area, cadence, stride duration, and stride length were all calculated as the mean gait characteristics. When passing through the glasswalk, the mouse’s paw area refers to the area of the mouse’s foot that touches the ground. Cadence is a unit of measurement for stride frequency, measured in steps per second. Stride duration (s) refers to the duration of one paw to complete a stride. The distance (cm) between successive placements of the same paw is known as the stride length. Behavioral characterization was approved by the ethical medical committee of Nanfang Hospital.

### Von Frey test

Mechanical allodynia was assessed using a digital electronic von Frey anesthesiometer, as previously described (IITC Life Science, CA, USA).^64,65^ This exam was performed five times with a 15 min inter-test delay between each repetition. Each mouse was hung above a wire grid in a clear plastic container. Before the trial, the animals were exposed to the testing environment for at least 15 min. The polypropylene non-Frey filament was placed perpendicular to the midplantar surface of a selected left hind paw. At the threshold, the mouse responded by flicking its paw away from the stimulus. The intensity of stimulation was determined when the mouse lifted its foot or added a paw.

### Flow cytometry

Following the dissection of soft tissue, the knee joints and blood were harvested. For the analysis of subchondral endothelial cells and MSCs, 10 subchondral bone specimens from 5 knee joints (including tibial and femoral subchondral bone in each knee joint) were used for cell isolation and analysis in each sample. We first removed the outer surface of the knee joint by immersing the specimens in protease solution (2.5 mg·mL^−1^ trypsin and 2 mg·mL^−1^ collagenase A) for 20 min. Then, the specimens were digested for 60 min to obtain the desired cells. Following the lysis of red blood cells (BD FACS™; BD Biosciences, San Jose, CA), we harvested cells within the supernatant, which were then used to detect the number of total endothelial cells (ECs), CD31^hi^Emcn^hi^ cells, and CD31^lo^Emcn^lo^ cells or to detect the alteration of MSCs. FACS was conducted with antibodies against CD31-APC (RD, FAB3628A-025), CD45-Per-CP (BioLegend, 103133), Ter119-Brilliant Violet 421 (BioLegend, 116233), Endomucin-FITC (Santa Cruz, sc-65495), Sca-1-PE (BioLegend, 108107), and CD24-Brilliant Violet 421 (BioLegend, 101825). CD31^hi^Emcn^hi^ cells were plotted and sorted by first setting standard quadrant gates. Then, as we previously described,^36^ in quadrant 2, gates were set arbitrarily at >10^3^ log Fl-2 (endomucin-FITC) and >10^3^ log Fl-4 (CD31-APC) fluorescence to discriminate CD31^hi^Emcn^hi^ cells from the total double-positive cells. After negative selection of the leukocyte common antigens CD119 and Ter45 at <10^2^ log Fl-3 (Ter119-Brilliant Violet 421) and <10^2^ log Fl-1 (CD45-Brilliant Violet 421), CD31^+^CD45^−^Ter119^−^, referred to as BMECs, were sorted using side scatter and CD31-APC fluorescence at >10^2^ log Fl-4 (CD31-APC). Cells were then resuspended in staining buffer and counted on an LSR II flow cytometer (BD Biosciences) before flow cytometry. CellQuest software was used to collect data on a FACSCalibur flow cytometer (Becton Dickinson). We used FlowJo software to analyze the data and create all flow cytometry contour plots (with outliers) (TreeStar).

### Quantitative real-time polymerase chain reaction analysis

The subchondral bones dissected from the proximal tibias, chilled in liquid nitrogen, were cut into pieces. We used TRIzol reagent (Invitrogen, Carlsbad, CA) for the homogenization of the specimens, and then, the total RNA was harvested. One milligram of total RNA was employed to synthesize cDNA using a cDNA Synthesis kit (Fermentas, Burlington, Canada). After that, FastStart Universal SYBR Premix ExTaqTM II (TaKaRa Biotechnology, Japan) was employed to conduct qRT‒PCR. The 2^−△△CT^ approach was employed to measure relative gene expression.

### Cell line and cell transfection

BMECs were cultured in DMEM (Gibco, USA) with 100 mg·mL^−1^ streptomycin sulfate (Life Technologies, USA), 15% fetal bovine serum (FBS; Gibco, USA), and 100 U·mL^−1^ penicillin. GENE provided the lentivirus used to construct PDGFR-β knockdown or overexpression constructs (Shanghai, China). PDGFR-β overexpression lentivirus (termed oePDGFR-β), a negative control (termed NC), PDGFR knockdown lentivirus (termed shPDGFR-β-7, shPDGFR-β-8, shPDGFR-β-9), or a scramble control (termed shNC) was used to infect BMECs plated in 6-well dishes at 50% confluence. Pools of stable transductions were formed using puromycin (7 μg·mL^−1^) and mycoplasma elimination reagent (0.1%, Yeasen, Shanghai) for 2 weeks. We carried out transfections employing the GENE transfection kit (GENE, China).

### LC–MS/MS analysis

BMECs were treated with PBS and PDGF-BB for 24 h. Then, we collected total cell lysates and incubated them with an antibody against PDGFR-β for immunoprecipitation. PDGFR-β-binding immunoprecipitates were subjected to LC–MS/MS analysis (PTM Bio, China). We converted the raw data (.wiff) into peak lists (.mgf) employing Protein Pilot software v4.0 (Applied Biosystems). Protein Pilot was employed to measure *P* values, the average relative expression, upper confidence interval, lower confidence interval, and error factors. Differential expression was defined as a fold change ≥ 1.5 or ≤0.5 and *P* values ≤ 0.05.

### Immunoprecipitation

Cells were washed two times with ice-cold PBS after treatment and lysed with ice-cold lysis buffer (0.3% CHAPS, 2 mmol·L^−1^ EDTA, 10 mmol·L^−1^ pyrophosphate, 10 mmol·L^−1^ glycerophosphate, 40 mmol·L^−1^ HEPES [pH 7.4], one tablet of EDTA-free protease inhibitors (Roche, USA) per 25 mL for 15 min. Then, we acquired the supernatant by centrifugation at 12 000 × *g* for 10 min. The supernatant was then incubated with primary antibody overnight at 4 °C. After that, a 50% slurry of Protein A + G Sepharose was added and incubated at 4 °C for another 2 h. The immunoprecipitates were washed 3 times with ice-cold PBS. Then, 50 µL of 1x SDS sample loading buffer was added, boiled for 10 min, and analyzed by immunoblotting. The following antibodies were used: PDGFR-β (Santa Cruz, 1:50, sc-374573), TLN1 (Abcam, 1:1 000, ab108480), FAK (CST, 1:1 000, 3285), goat anti-mouse IgG HRP (Invitrogen, 1:10 000, 31430), and goat anti-rabbit IgG HRP (Invitrogen, 1:10 000, 31460).

### Western blot assay

PDGFR-β proteins were collected from subchondral bone tissue. The bone tissue was ground in liquid nitrogen until it became a powder. The cell proteins were collected from BMECs. Cell lysis buffer was added to extract the subchondral bone or BMEC protein. The PierceTM BCA Protein Assay Kit was employed to determine the protein levels (Thermo Fisher Scientific, Waltham, MA). Next, 1x SDS sample loading buffer was added to the cell lysates and boiled for 10 min. After protein extraction, the supernatants were separated by sodium dodecyl sulfate‐polyacrylamide gel electrophoresis (Yamei, PG110-114) and blotted onto polyvinylidene fluoride membranes. After the membranes were blocked with skim milk, the antibodies were added to the primary antibody dilution and incubated with the membranes overnight at 4 °C. We washed the membranes with TBST three times (10 min each time). Then, goat anti-mouse IgG (1:10 000) or goat anti-rabbit IgG (1:10 000) was added and incubated at room temperature for 60 min. After that, we washed the membranes three times again (10 min each time). Enhanced chemiluminescence (ECL Kit; Amersham Biosciences) was used for protein visualization. The following Western blotting antibodies and inhibitors were used: PDGFR-β (Santa Cruz, 1:1 000, sc-374573), phospho-FAK (Tyr925) (CST, 1:1 000, 3284), phospho-FAK (Tyr397) (CST, 1:1 000, 3283), FAK (CST, 1:1 000, 3285), TLN1 (CST, 1:1 000, 4021), phospho-TLN1 (Ser425) (CST, 1:1 000, 5426), VEGF (Proteintech, 1:1 000, 19003-1-AP), goat anti-mouse IgG HRP (Invitrogen, 1:10 000, 31430), goat anti-rabbit IgG HRP (Invitrogen, 1:10 000, 31460) and PF-573228 (MCE, 50 nmol·L^−1^, HY-10461).

### Enzyme-linked immunosorbent assay (ELISA)

We cultured cells in DMEM with 15% FBS for 24 h and collected the cell culture medium by centrifugation at 200 × *g* for 15 min. All samples were stored at −80 °C before analyses. Next, the supernatant from various treatment conditions was collected to quantify VEGF levels employing a commercial ELISA kit (ab100786; Abcam) (five per group). The absorbance of the ELISAs listed above was measured by employing a microplate reader at 450 nm (Bio-Rad 680, Hercules, USA). The wavelength was corrected to 570 nm. The protein concentration in each sample was calculated using the standard curve.

### Tube formation assay

BMECs at a density of 2 × 10^4^ cells per well were seeded in 96-well plates that were already coated with 50 μL of Matrigel in each seed well (BD Biosciences). The plates were then incubated at 37 °C under different treatment conditions. After culture for 3 h, the cells were imaged and analyzed using an inverted microscope (Leica) and Image-Pro Plus 6 software. Total loops, total branching points, and total tube length were measured.

### Cell proliferation assay

A Cell Counting Kit-8 was employed to detect BMEC proliferation (CCK-8; Dojindo, Kumamoto, Japan). In brief, BMECs at a density of 3 000 cells per well were seeded in a 96-well plate and incubated in DMEM under different treatments for 24 h at 37 °C. The wells treated with complete culture medium (without cells) were set as blanks. We added 10 μL of CCK-8 solution to each well on Days 1, 3, and 5. In addition, the wells were incubated at 37 °C for 2 h. The optical density (OD) was detected at 450 nm based on a microplate reader (Bio-Rad 680). The cell proliferation rate was evaluated by drawing a growth curve.

### Migration assay

The wound scratch assay was used to analyze the migration of BMECs under different conditions. In brief, 5.0 × 10^5^ cells per well were plated in a 35 mm culture plate and incubated for 24 h at 37 °C until confluence. Next, a scratch was made with a sterile p200 pipette tip. Cell fragments were washed 3 times with PBS and then incubated in DMEM with 5% fetal bovine serum (FBS; Gibco, USA) under different treatments. Images of the wounds were taken immediately, 12 h, and 24 h after scratching. We used ImageJ software to detect the change in the width of the scratched areas. The rate of migration was measured as follows: migration area (%) = (A0 – An)/A0 × 100, where An and A0 represent the residual area and initial area of the wound, respectively.

### Statistical analysis

Data are presented as the mean ± s.d. For two-group comparisons, data were analyzed by a two-tailed Student’s *t*-test. For multiple group comparisons, one-way analysis of variance (ANOVA) was used. We first examined homogeneity of variance and then evaluated the differences between groups using post hoc multiple comparisons. Specifically, we used Dunnett’s T3 to evaluate the group differences if heterogeneity existed. However, the Bonferroni test was adopted if there was no heterogeneity. Significant differences were defined at *P* < 0.05. SPSS 22.0 analysis software (SPSS, Inc.) was employed for all data analyses.

## Supplementary information

Editing certificate by Springer nature

Supplementary data--original images of IF staining

Supplementary figure legends-clean version

Supplementary figure legends-marked up

Supplementary figure 1

Supplementary figure 2

Supplementary figure 3

supplementary figure4

supplementary figure5

Supplementary figure 6

supplementary figure 7

Supplementary figure 8

Supplementary figure 9

supplementary figure 10

supplementary figure 11

Supplementary Table 1

## References

[1] Zhen G (2021). Mechanical stress determines the configuration of TGFβ activation in articular cartilage. Nat. Commun..

[2] Hootman JM, Helmick CG, Barbour KE, Theis KA, Boring MA (2016). Updated projected prevalence of self-reported doctor-diagnosed arthritis and arthritis-attributable activity limitation among US adults, 2015-2040. Arthritis Rheumatol..

[3] Hu Y, Chen X, Wang S, Jing Y, Su J (2021). Subchondral bone microenvironment in osteoarthritis and pain. Bone Res..

[4] Lories RJ, Luyten FP (2011). The bone-cartilage unit in osteoarthritis. Nat. Rev. Rheumatol..

[5] Burr DB, Gallant MA (2012). Bone remodelling in osteoarthritis. Nat. Rev. Rheumatol..

[6] Zhen G (2013). Inhibition of TGF-beta signaling in mesenchymal stem cells of subchondral bone attenuates osteoarthritis. Nat. Med..

[7] Qin H (2019). SDF-1/CXCR4 axis coordinates crosstalk between subchondral bone and articular cartilage in osteoarthritis pathogenesis. Bone.

[8] Chen D (2017). Osteoarthritis: toward a comprehensive understanding of pathological mechanism. Bone Res..

[9] Brandt KD, Radin EL, Dieppe PA, van de Putte L (2006). Yet more evidence that osteoarthritis is not a cartilage disease. Ann. Rheum. Dis..

[10] Tuckermann J, Adams RH (2021). The endothelium-bone axis in development, homeostasis and bone and joint disease. Nat. Rev. Rheumatol..

[11] Huang J (2018). Harmine enhances type H vessel formation and prevents bone loss in ovariectomized mice. Theranostics.

[12] Portal-Núñez S, Lozano D, Esbrit P (2012). Role of angiogenesis on bone formation. Histol. Histopathol..

[13] Cleaver O, Melton DA (2003). Endothelial signaling during development. Nat. Med..

[14] Chim SM (2013). Angiogenic factors in bone local environment. Cytokine Growth Factor. Rev..

[15] Brandi ML, Collin-Osdoby P (2006). Vascular Biology and the Skeleton. J. Bone Miner. Res..

[16] Kusumbe AP, Ramasamy SK, Adams RH (2014). Coupling of angiogenesis and osteogenesis by a specific vessel subtype in bone. Nature.

[17] Ramasamy SK, Kusumbe AP, Wang L, Adams RH (2014). Endothelial Notch activity promotes angiogenesis and osteogenesis in bone. Nature.

[18] Cui Z (2016). Halofuginone attenuates osteoarthritis by inhibition of TGF-β activity and H-type vessel formation in subchondral bone. Ann. Rheum. Dis..

[19] Hu Y (2020). Defactinib attenuates osteoarthritis by inhibiting positive feedback loop between H-Type vessels and MSCs in subchondral bone. J. Orthop. Transl..

[20] Andrae J, Gallini R, Betsholtz C (2008). Role of platelet-derived growth factors in physiology and medicine. Genes. Dev..

[21] Rolny C (2006). Platelet-derived growth factor receptor-beta promotes early endothelial cell differentiation. Blood.

[22] Caplan AI, Correa D (2011). PDGF in bone formation and regeneration: new insights into a novel mechanism involving MSCs. J. Orthop. Res..

[23] Battegay EJ, Rupp J, Iruela-Arispe L, Sage EH, Pech M (1994). PDGF-BB modulates endothelial proliferation and angiogenesis in vitro via PDGF beta-receptors. J. Cell. Biol..

[24] Xie H (2014). PDGF-BB secreted by preosteoclasts induces angiogenesis during coupling with osteogenesis. Nat. Med..

[25] Gao B (2019). Macrophage-lineage TRAP^+^ cells recruit periosteum-derived cells for periosteal osteogenesis and regeneration. J. Clin. Invest..

[26] Rindone AN (2021). Quantitative 3D imaging of the cranial microvascular environment at single-cell resolution. Nat. Commun..

[27] Zhen G (2021). An antibody against Siglec-15 promotes bone formation and fracture healing by increasing TRAP+ mononuclear cells and PDGF-BB secretion. Bone Res..

[28] Santhanam L (2021). Skeleton-secreted PDGF-BB mediates arterial stiffening. J. Clin. Invest..

[29] Su W (2020). Angiogenesis stimulated by elevated PDGF-BB in subchondral bone contributes to osteoarthritis development. JCI. insight.

[30] Clarke J (2020). PDGF-BB is the key to unlocking pathological angiogenesis in OA. Nat. Rev. Rheumatol..

[31] Liu T (2018). PDGF-mediated mesenchymal transformation renders endothelial resistance to anti-VEGF treatment in glioblastoma. Nat. Commun..

[32] Critchley DR (2009). Biochemical and structural properties of the integrin-associated cytoskeletal protein talin. Annu. Rev. Biophys..

[33] Ratnikov B (2005). Talin phosphorylation sites mapped by mass spectrometry. J. Cell. Sci..

[34] Monkley SJ (2011). Endothelial cell talin1 is essential for embryonic angiogenesis. Dev. Biol..

[35] Pulous FE (2021). Talin-dependent integrin activation is required for endothelial proliferation and postnatal angiogenesis. Angiogenesis.

[36] Yang M (2017). MiR-497~195 cluster regulates angiogenesis during coupling with osteogenesis by maintaining endothelial Notch and HIF-1a activity. Nat. Commun..

[37] Pritzker KP (2006). Osteoarthritis cartilage histopathology: grading and staging. Osteoarthr. Cartil..

[38] Roemer FW (2009). Change in MRI-detected subchondral bone marrow lesions is associated with cartilage loss: the MOST Study. A longitudinal multicentre study of knee osteoarthritis. Ann. Rheum. Dis..

[39] Fu S, Wang C, Yang R, Wu F, Hsiao F (2017). Bisphosphonate use and the risk of undergoing total knee arthroplasty in osteoporotic patients with osteoarthritis: a nationwide cohort study in Taiwan. J. Bone Jt. Surg. Am..

[40] Hu B (2020). Sensory nerves regulate mesenchymal stromal cell lineage commitment by tuning sympathetic tones. J. Clin. Invest..

[41] Ambrosi TH (2017). Adipocyte accumulation in the bone marrow during obesity and aging impairs stem cell-based hematopoietic and bone regeneration. Cell. Stem. Cell..

[42] Lawson C (2012). FAK promotes recruitment of talin to nascent adhesions to control cell motility. J. Cell. Biol..

[43] Cheng L (2020). Clinically relevant high levels of human C-reactive protein induces endothelial dysfunction and hypertension by inhibiting the AMPK-eNOS axis. Clin. Sci..

[44] Varadi K (2012). Novel random peptide libraries displayed on AAV serotype 9 for selection of endothelial cell-directed gene transfer vectors. Gene. Ther..

[45] Findlay DM, Kuliwaba JS (2016). Bone-cartilage crosstalk: a conversation for understanding osteoarthritis. Bone Res..

[46] Hunter DJ, Felson DT (2006). Osteoarthritis. BMJ.

[47] Meachim G (1963). The effect of scarification on articular cartilage in the rabbit. J. Bone Jt. Surg. Br..

[48] Neogi T, Li S, Peloquin C, Misra D, Zhang Y (2018). Effect of bisphosphonates on knee replacement surgery. Ann. Rheum. Dis..

[49] Raica M, Cimpean AM (2010). Platelet-Derived Growth Factor (PDGF)/PDGF Receptors (PDGFR) axis as target for antitumor and antiangiogenic therapy. Pharmaceuticals.

[50] Dubrac A (2018). NCK-dependent pericyte migration promotes pathological neovascularization in ischemic retinopathy. Nat. Commun..

[51] Krasnokutsky S (2011). Quantitative magnetic resonance imaging evidence of synovial proliferation is associated with radiographic severity of knee osteoarthritis. Arthritis Rheum..

[52] Yusuf E, Kortekaas MC, Watt I, Huizinga TW, Kloppenburg M (2011). Do knee abnormalities visualised on MRI explain knee pain in knee osteoarthritis? A systematic review. Ann. Rheum. Dis..

[53] Zhu S (2019). Subchondral bone osteoclasts induce sensory innervation and osteoarthritis pain. J. Clin. Invest..

[54] Kopp PM (2010). Studies on the morphology and spreading of human endothelial cells define key inter- and intramolecular interactions for talin1. Eur. J. Cell. Biol..

[55] Senetar MA, McCann RO (2005). Gene duplication and functional divergence during evolution of the cytoskeletal linker protein talin. Gene.

[56] Zhang X (2008). Talin depletion reveals independence of initial cell spreading from integrin activation and traction. Nat. Cell. Biol..

[57] Monkley SJ, Pritchard CA, Critchley DR (2001). Analysis of the mammalian talin2 gene TLN2. Biochem. Biophys. Res. Commun..

[58] Debrand E (2009). Talin 2 is a large and complex gene encoding multiple transcripts and protein isoforms. FEBS. J..

[59] Rui YN (2017). The Intracranial Aneurysm Gene THSD1 connects endosome dynamics to nascent focal adhesion assembly. Cell. Physiol. Biochem..

[60] Chen H (2019). Prostaglandin E2 mediates sensory nerve regulation of bone homeostasis. Nat. Commun..

[61] Walsh DA (2010). Angiogenesis and nerve growth factor at the osteochondral junction in rheumatoid arthritis and osteoarthritis. Rheumatology.

[62] Zhou C (2021). Runx1 protects against the pathological progression of osteoarthritis. Bone Res..

[63] Nyul-Toth A (2021). Early manifestation of gait alterations in the Tg2576 mouse model of Alzheimer’s disease. GeroScience.

[64] Ding R (2016). Advanced oxidation protein products sensitized the transient receptor potential vanilloid 1 via NADPH oxidase 1 and 4 to cause mechanical hyperalgesia. Redox Biol..

[65] Toussaint AB (2022). Chronic paternal morphine exposure increases sensitivity to morphine-derived pain relief in male progeny. Sci. Adv..

