# Supplementary material for: Endothelial PDGF-BB/PDGFR-β signaling promotes osteoarthritis by enhancing angiogenesis-dependent abnormal subchondral bone formation
Source: Bone Res. 2022 Aug 29;10:58. doi: 10.1038/s41413-022-00229-6 (PMC9420732; doi:10.1038/s41413-022-00229-6)
Supplement: Supplementary file 1 — Editing certificate by Springer nature [file 41413_2022_229_MOESM1_ESM.pdf]

SPRINGER NATURE

Author Services

Editing Certificate

This document certifies that the manuscript

Endothelial PDGF-BB/PDGFR-β signaling promotes osteoarthritis by enhancing angiogenesis-dependent abnormal subchondral bone formation

prepared by the authors

Zhuang Cui

was edited for proper English language, grammar, punctuation, spelling, and overall style by one or more of the highly qualified native English speaking editors at SNAS.

This certificate was issued on **March 30, 2022** and may be verified on the SNAS website using the verification code **9D25-1C27-C123-8D7A-E524**.

Neither the research content nor the authors' intentions were altered in any way during the editing process. Documents receiving this certification should be English-ready for publication; however, the author has the ability to accept or reject our suggestions and changes. To verify the final SNAS edited version, please visit our verification page at secure.authorservices.springernature.com/certificate/verify.
If you have any questions or concerns about this edited document, please contact SNAS at support@as.springernature.com.

SNAS provides a range of editing, translation, and manuscript services for researchers and publishers around the world.
For more information about our company, services, and partner discounts, please visit authorservices.springernature.com.
