# Supplementary material for: Endothelial PDGF-BB/PDGFR-β signaling promotes osteoarthritis by enhancing angiogenesis-dependent abnormal subchondral bone formation
Source: Bone Res. 2022 Aug 29;10:58. doi: 10.1038/s41413-022-00229-6 (PMC9420732; doi:10.1038/s41413-022-00229-6)

Figure 2

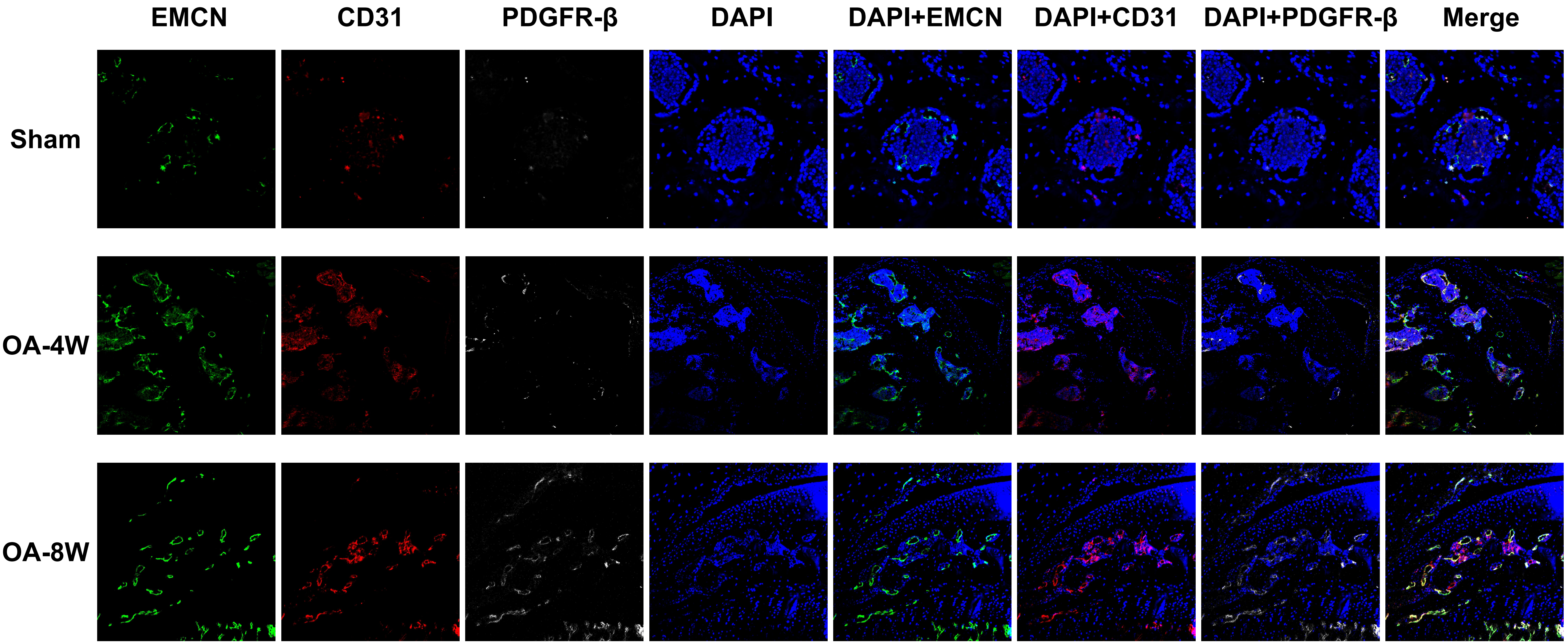

EMCN
CD31
PDGFR-β
DAPI
DAPI+EMCN
DAPI+CD31
DAPI+PDGFR-β
Merge
Sham
OA-4W
OA-8W

Figure 4

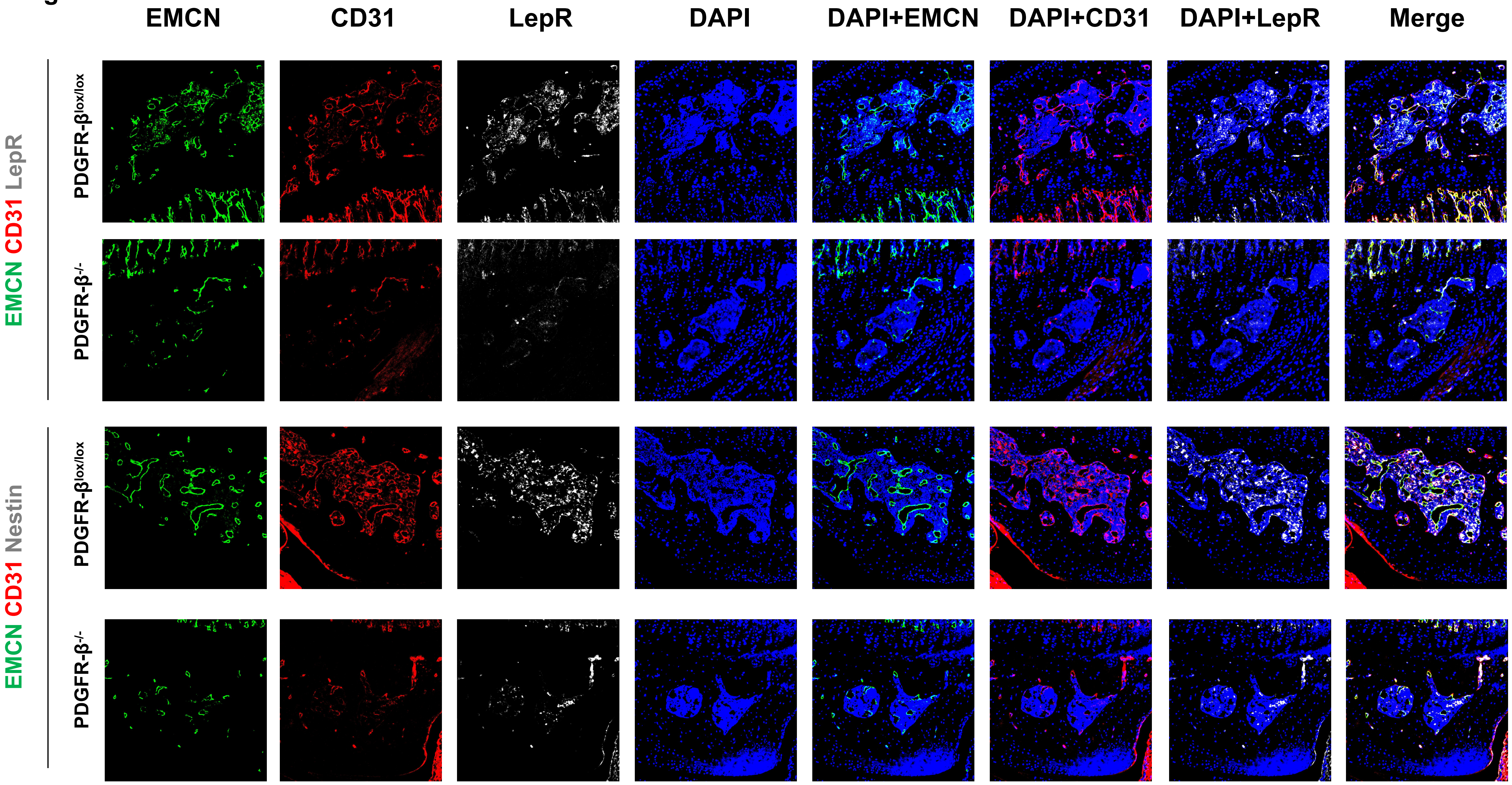

EMCN
CD31
LepR
DAPI
DAPI+EMCN
DAPI+CD31
DAPI+LepR
Merge
EMCN CD31 LepR
PDGFR-β^lox/lox
PDGFR-β^-/-
EMCN CD31 Nestin
PDGFR-β^lox/lox
PDGFR-β^-/-

Figure 7

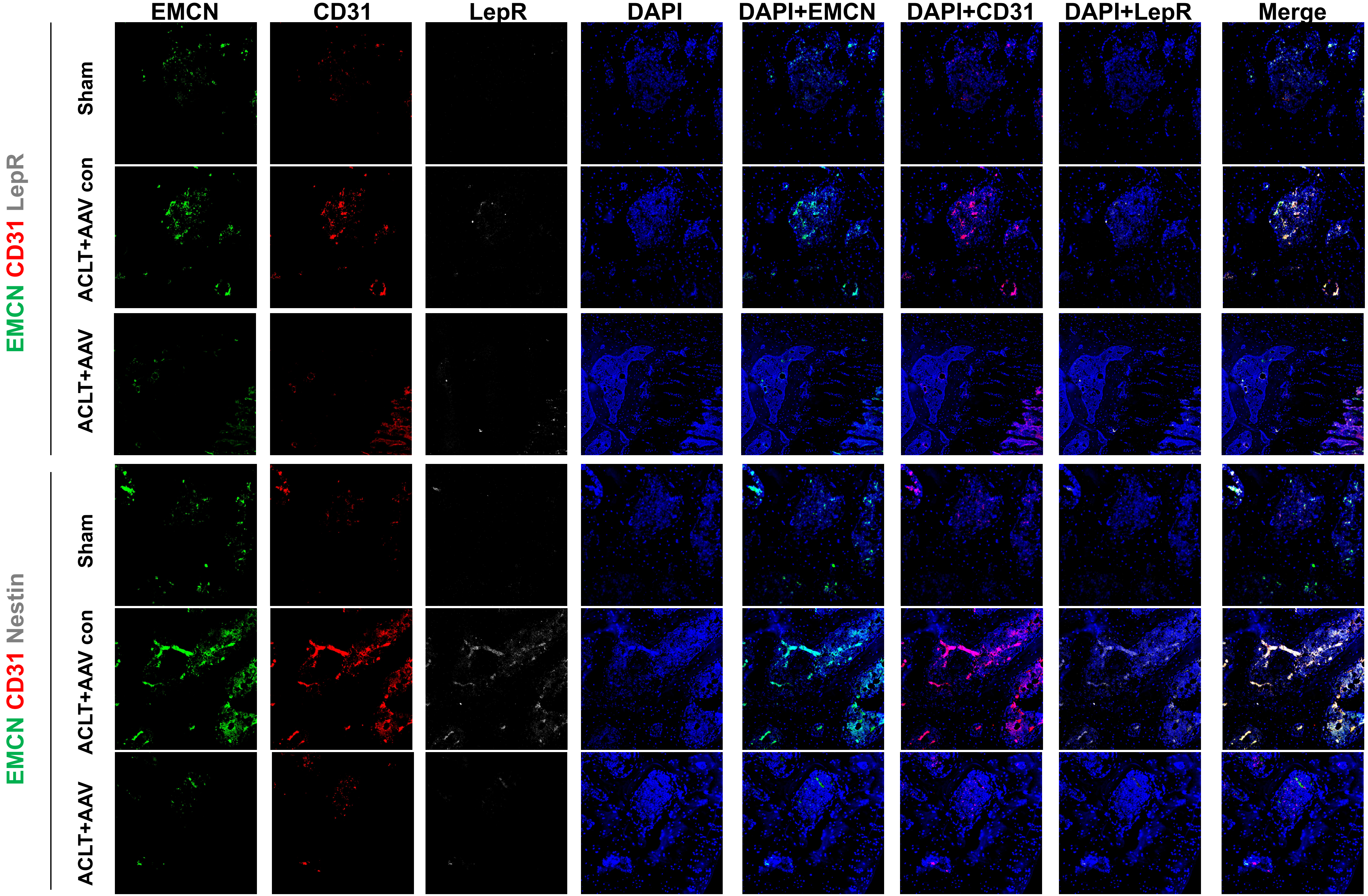
EMCN
CD31
LepR
DAPI
DAPI+EMCN
DAPI+CD31
DAPI+LepR
Merge
EMCN CD31 LepR
Sham
ACLT+AAV con
ACLT+AAV
EMCN CD31 Nestin
Sham
ACLT+AAV con
ACLT+AAV

Supplemental figure 4

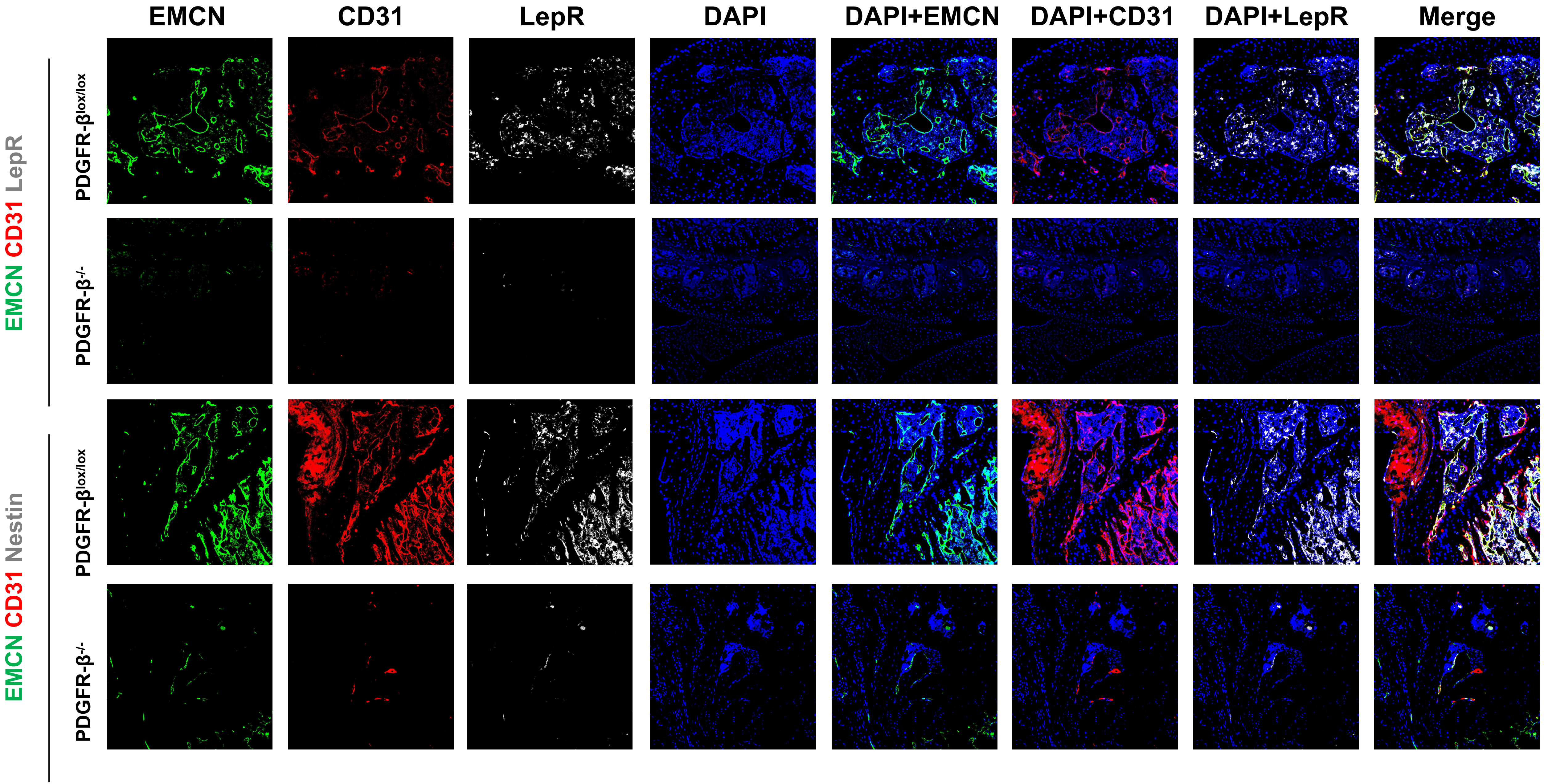

Supplemental figure 5 2M

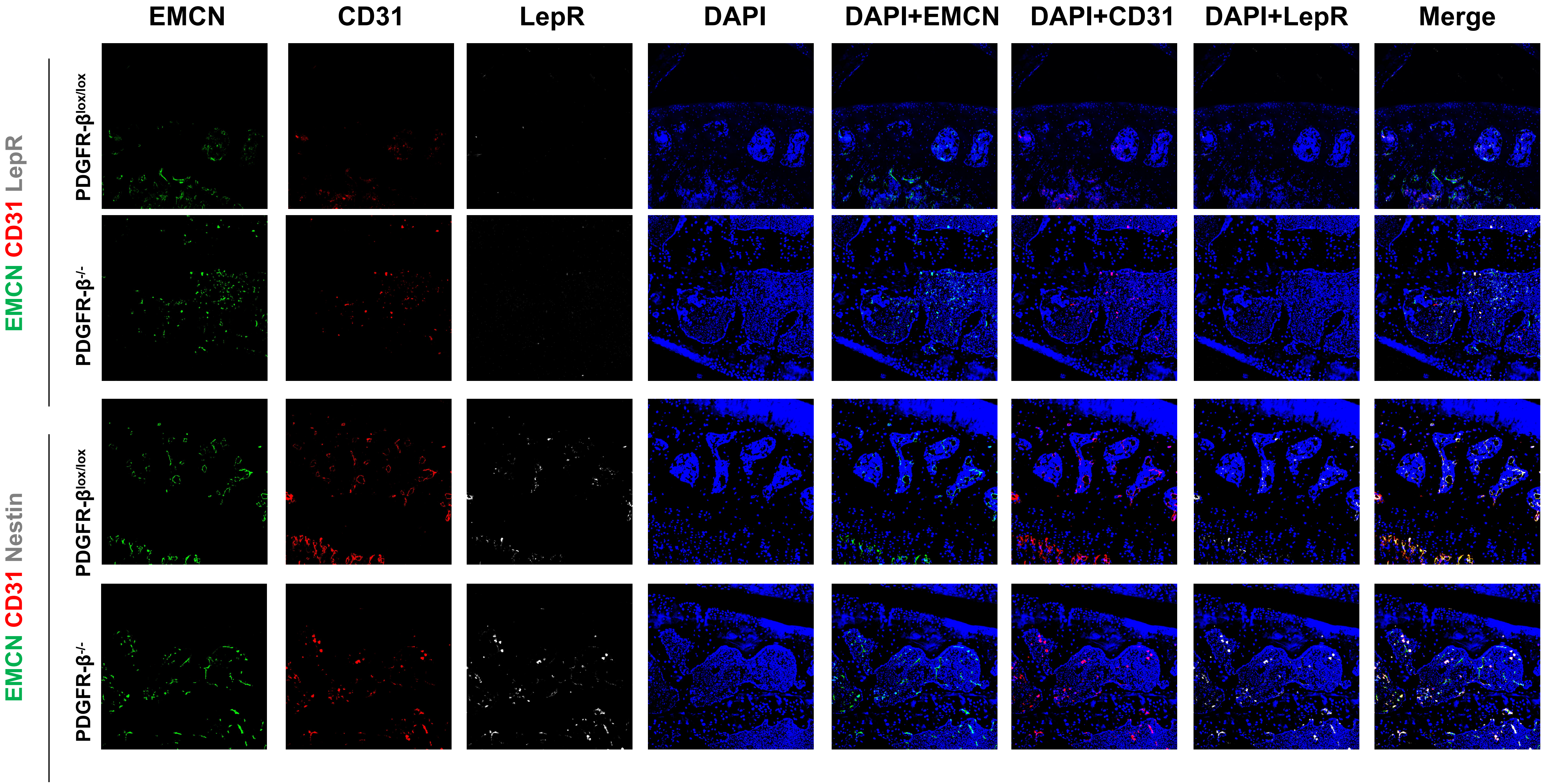

Supplemental figure 5 15M

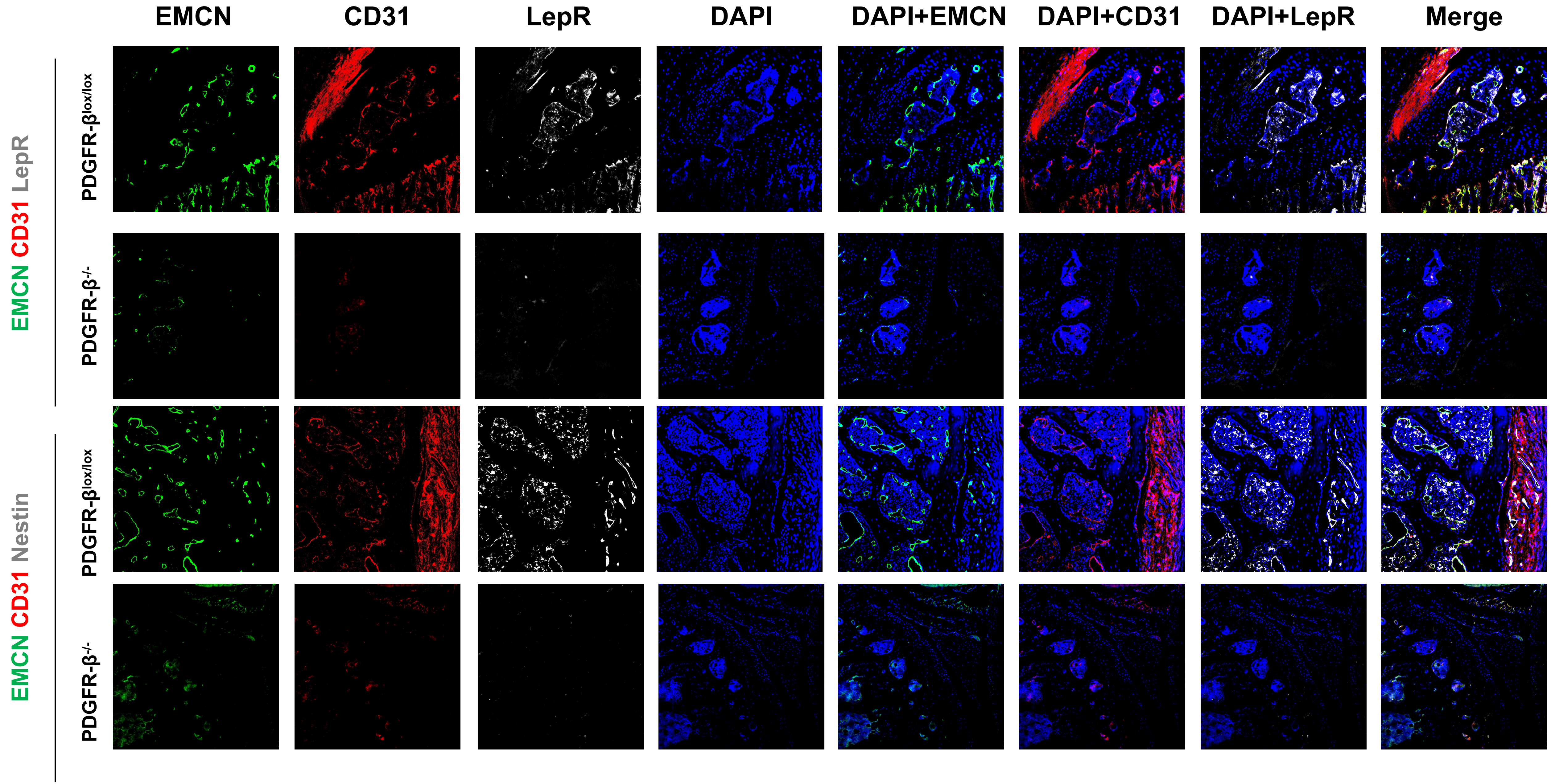

Supplement: Supplementary file 2 — Supplementary data--original images of IF staining [file 41413_2022_229_MOESM2_ESM.pdf]
