# Supplementary material for: Endothelial PDGF-BB/PDGFR-β signaling promotes osteoarthritis by enhancing angiogenesis-dependent abnormal subchondral bone formation
Source: Bone Res. 2022 Aug 29;10:58. doi: 10.1038/s41413-022-00229-6 (PMC9420732; doi:10.1038/s41413-022-00229-6)
Supplement: Supplementary file 3 — Supplementary figure legends-clean version [file 41413_2022_229_MOESM3_ESM.docx]

**Supplementary figure legends**

**Supplemental Figure 1** **Alterations in PDGFR-β levels, subchondral bone and articular cartilage in aged mice**. (a) Immunostaining of PDGFR-β-positive cells (upper) in the subchondral bone of mice with the magnified area (bottom) in the boxed area in the upper image of the mice at 1, 6 and 15 months of age. Scale bar, top 100 µm; bottom 50 µm. (b) Quantification of PDGFR-β-positive cells in the subchondral bone of the mice at 1, 6 and 15 months of age. n = 10. (c) µCT images of the subchondral bone medial compartment of the mice at 1, 6 and 15 months of age. Scale bar, top 500 µm. (d-g) Quantitative analysis of the bone tissue relative to BV/TV (bone tissue relative to the total tissue volume) (d), Tb.pf (trabecular pattern factor) (e), SBP.Th (subchondral bone plate thickness) (f) and Tb.N (trabecular N) (g) in the mice at 1, 6 and 15 months of age. n = 10. (h) SOFG (upper) with the magnified area (bottom) in the boxed area in the upper image; proteoglycan (red) and bone (green). Scale bar, top 100 µm; bottom 50 µm. (i) OARSI-modified Mankin scores of articular cartilage in the mice at 1, 6 and 15 months of age. n = 10. *p < 0.05 and **p <0 .01 as compared to the sham group or as denoted by bars.

**Supplemental Figure 2 The basic characteristics of mice with the deletion of PDGFR-β in ECs.** (a) qRT-PCR examination of the PDGFR-β in the subchondral bone from the PDGFR-β^lox/lox^ and PDGFR-β^-/-^ mice. n = 5. (b-d) Gross inspection of the PDGFR-β^lox/lox^ and PDGFR-β^-/-^ mice (b) and quantitative analysis of the alterations in body weight (c) and leg length (d). n = 8 per group. *p < 0.05 and **p <0 .01 as compared to the sham group or as denoted by bars.

**Supplemental Figure 3** **Articular cartilage degeneration is attenuated in aged mice with PDGFR-β deletion in ECs**. (a) SOFG (upper) with the magnified area (bottom) in the boxed area in the upper image of the PDGFR-β^lox/lox^ and PDGFR-β^-/-^ mice at 2 and 15 months of age; proteoglycan (red) and bone (green). H&E staining with hyaline cartilage (HC) and calcified cartilage (CC) are separated by black dashed lines in the PDGFR-β^lox/lox^ and PDGFR-β^-/-^ mice at 2 and 15 months of age. Scale bar, top 100 µm; middle 50 µm; bottom 50 µm. (b and c) Quantitative analysis of OARSI-modified Mankin scores (b) and the ratio of hyaline cartilage relative to the calcified cartilage (HC/CC) (c) in the PDGFR-β^lox/lox^ and PDGFR-β^-/-^ mice at 2 and 15 months of age. n = 8. *p < 0.05 and **p <0 .01 as compared to the PDGFR-β^lox/lox^ controls or as denoted by bars.

**Supplemental Figure 4 Alterations in H-type vessels and their linked MSCs in the subchondral bone of mice with PDGFR-β deletion in ECs.** (a) Immunostaining of PDGFR-β-positive cells in the subchondral bone of the PDGFR-β^lox/lox^ and PDGFR-β^-/-^ mice at pre- and post-ACLT. Scale bar, 100 µm. (b) Quantification of PDGFR-β-positive cells in the subchondral bone of the PDGFR-β^lox/lox^ and PDGFR-β^-/-^ mice at pre- and post-ACLT. n = 8. (c) Confocal images (upper) with the magnified area (bottom) in the boxed area in the upper image of H-type vessels (CD31: red; endomucin: green; merge: yellow) and LepR^+^ cells (left part, white), and Nestin^+^ cells (right part, white) in the subchondral bone from the PDGFR-β^lox/lox^ and PDGFR-β^-/-^ mice at 8 weeks post-ACLT. Scale bar, top 100 µm; bottom 50 µm. (d-f) Quantification of H-type vessel volume(d), LepR^+^ cells(e) and Nestin^+^ cells (f) in the subchondral bone from the PDGFR-β^lox/lox^ and PDGFR-β^-/-^ mice at 8 weeks post-ACLT. n = 8. *p < 0.05 and **p <0 .01 as compared to the PDGFR-β^lox/lox^ controls or as denoted by bars.

**Supplemental Figure 5** **Alteration in H-type vessels and their linked MSCs in the subchondral bone of aged PDGFR-β deletion mice.** (a) Confocal images (upper) with the magnified area (bottom) in the boxed area in the upper image of H-type vessels (CD31: red; endomucin: green; merge: yellow) and LepR^+^ cells (left part, white), and Nestin^+^ cells (right part, white) in the subchondral bone from the PDGFR-β^lox/lox^ and PDGFR-β^-/-^ mice at 2 and 15 months of age. Scale bar, top 100 µm; bottom 50 µm. (b-d) Quantification of H-type vessel volume(b), LepR^+^ cells(c) and Nestin^+^ cells (d) in the subchondral bone from the PDGFR-β^lox/lox^ and PDGFR-β^-/-^ mice at 2 and 15 months of age. n = 8. *p < 0.05 and **p <0 .01 as compared to the PDGFR-β^lox/lox^ controls or as denoted by bars.

**Supplemental Figure 6 Alterations in Catwalk analysis of the PDGFR-β^lox/lox^ and PDGFR-β^-/-^ mice with OA.** (a) Representative images of Catwalk analysis of PDGFR-β^lox/lox^ and PDGFR-β^-/-^ mice at 4 weeks and 8 weeks post-ACLT surgery on the left knees. RH: right hind; LH: left hind. (b-e) Quantitative analysis of the gait data relative to the area of the mouse's left hind that touches the ground (left hind paw area) (b); left hind stride frequency (cadence) (c); the time elapsed for the subsequent left hind touch (stride duration) (d); the distance between successive placements of the left hind (stride length) (e). n = 8. *p < 0.05 and **p <0 .01 as compared to the PDGFR-β^lox/lox^ controls or as denoted by bars.

**Supplementary figure 7 The paw withdrawal threshold of PDGFR-β^-/-^ and PDGFR-β^lox/lox^ mice in OA.** (a) The electronic Von Frey anaesthesiometer system and experimental schematic diagram. (b) Paw mechanical threshold was tested by the electronic Von Frey system from PDGFR-β^lox/lox^ and PDGFR-β^-/-^ mice at 4 and 8 weeks after ACLT surgery. PDGFR-β knockdown improved the paw withdrawal threshold in a significant manner compared with the PDGFR-β^lox/lox^ controls at 4 and 8 weeks after ACLT surgery. n = 8. *p < 0.05 and **p <0 .01 as compared to PDGFR-β^lox/lox^ controls or as denoted by bar.

**Supplemental Figure 8 Rat model of injection of the AAV into subchondral bone.** (a and b) A strategy to inject AAV into subchondral bone in rats. (c) Immunofluorescence (IF) of GFP expression in subchondral bone tissue after AAV control and AAV9 administration. IF data showed GFP expression in the vessels of subchondral bone of knee joints of SD rat (upper) with the magnified area (bottom) in the boxed area in the upper image of animals that received AAV control and AAV silencing pdgfrb injections at the age of 3 months. Both the AAV-control and AAV9 groups expressed GFP as a reporter of AAV9 infection efficiency. n = 5. Scale bar, top 100 µm; bottom 50 µm.

**Supplemental Figure 9** **Local specific inhibition of PDGFR-β in the subchondral bone attenuates articular cartilage degeneration in rats.** (a-f) Immunostaining (a) and quantification of MMP-13 (b), Sox9 (c), ADAMTS 5 (d), Col II (e) and Aggrecan (f) in the articular cartilage from the sham, ACLT + AAV control and ACLT + AAV rats at 8 weeks post-ACLT. n = 5. Scale bar, 50 µm. Sham= sham controls; ACLT+AAV control = AAV control treated ACLT rats; ACLT +AAV = AAV for silencing endothelial PDGFR-β treated ACLT rats; *p < .05 and **p < .01 as compared to the sham group. ^#^p < .05 and ^##^p < .01 as compared to the AAV control group.

**Supplemental Figure 10 and 11 Whole gel images for western blots in figure 5**

Blue dotted boxes showed the bands were from the same blot. The bands labeled with red color indicated that the bands were shown in the figures. The bands labeled with yellow color were not shown in the figures.

.
