# Supplementary figures and images for: Endothelial PDGF-BB/PDGFR-β signaling promotes osteoarthritis by enhancing angiogenesis-dependent abnormal subchondral bone formation
Source: Bone Res. 2022 Aug 29;10:58. doi: 10.1038/s41413-022-00229-6 (PMC9420732; doi:10.1038/s41413-022-00229-6)

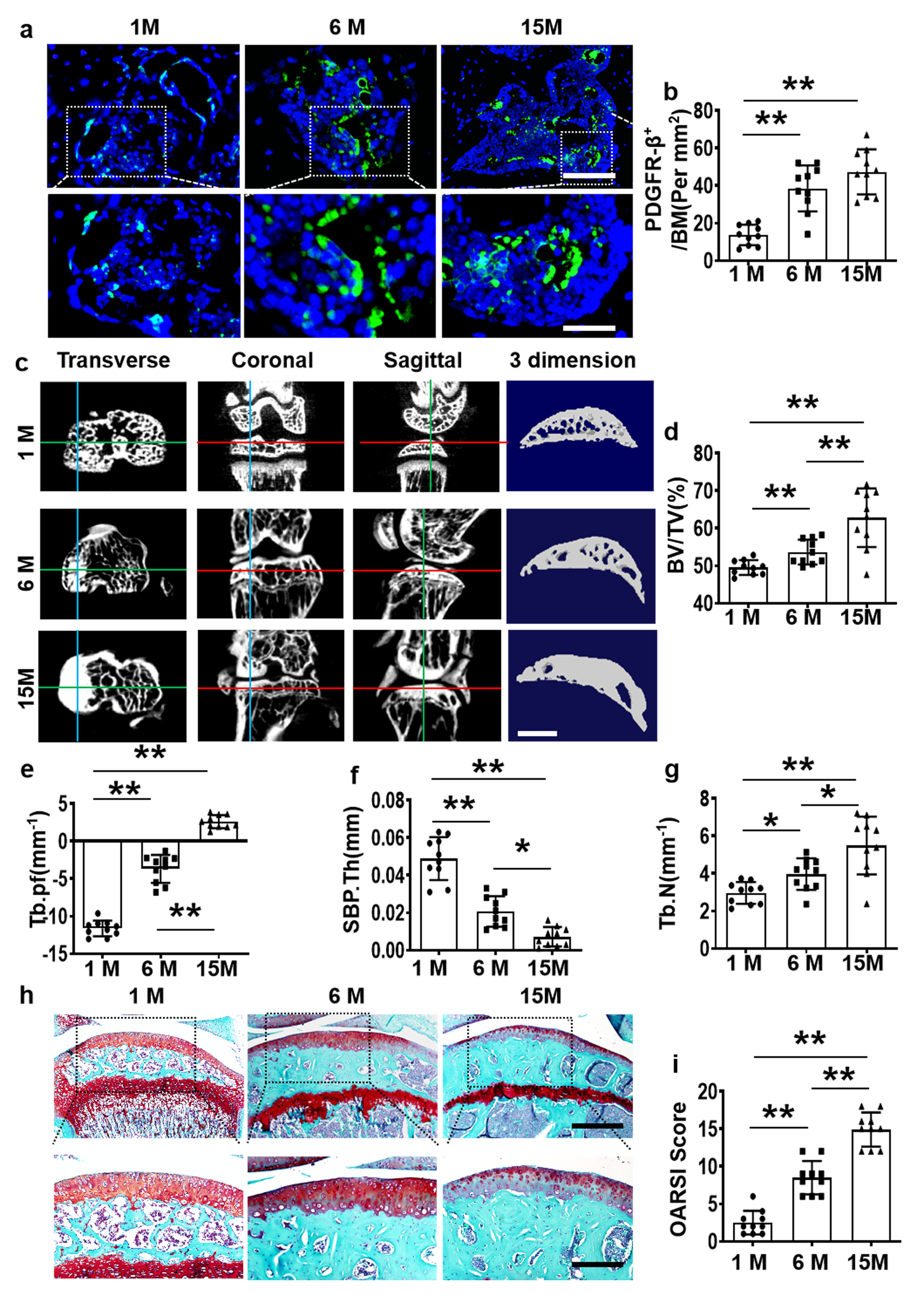

Supplement: Supplementary file 5 — Supplementary figure 1 [file 41413_2022_229_MOESM5_ESM.tif]

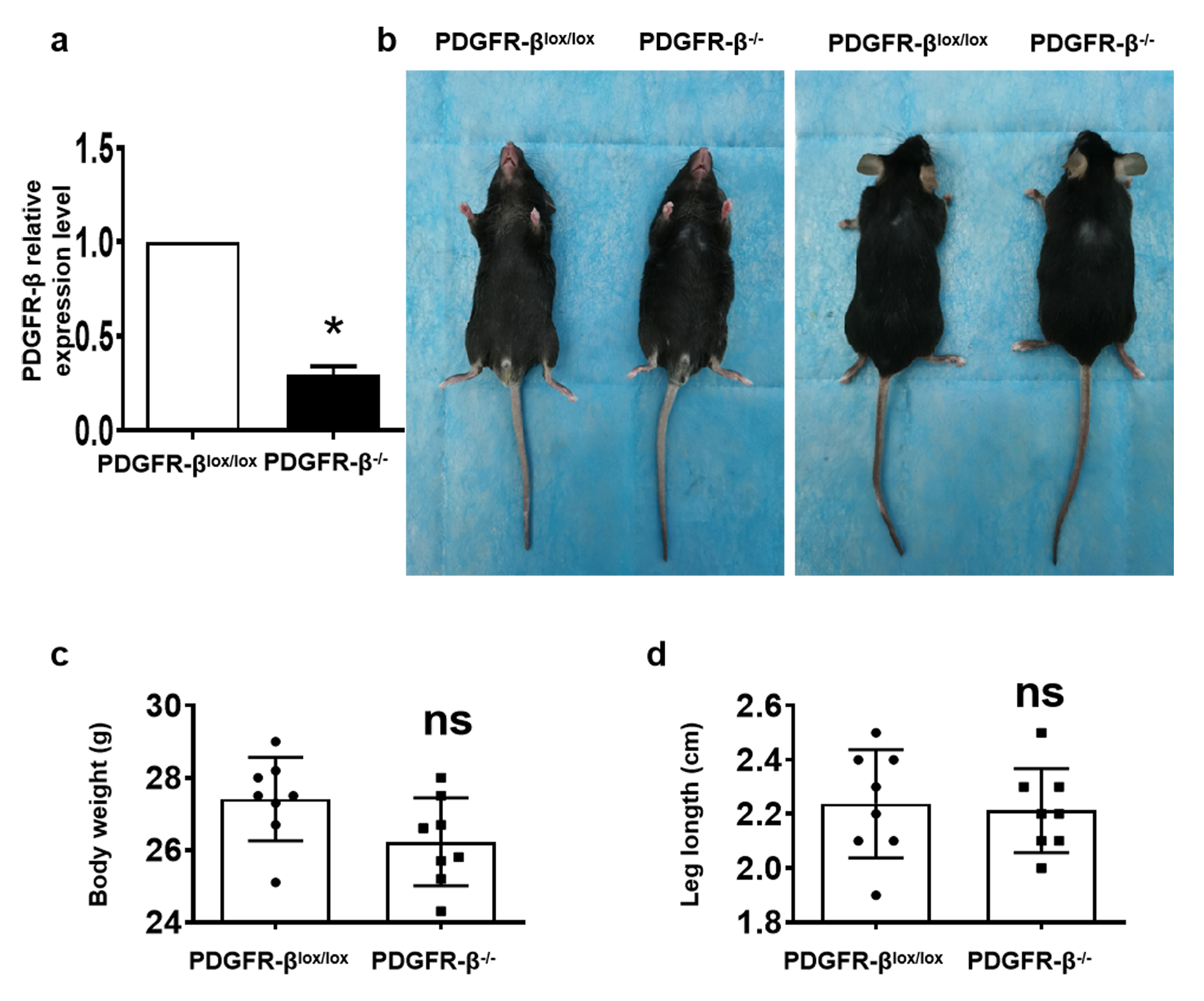

Supplement: Supplementary file 6 — Supplementary figure 2 [file 41413_2022_229_MOESM6_ESM.tif]

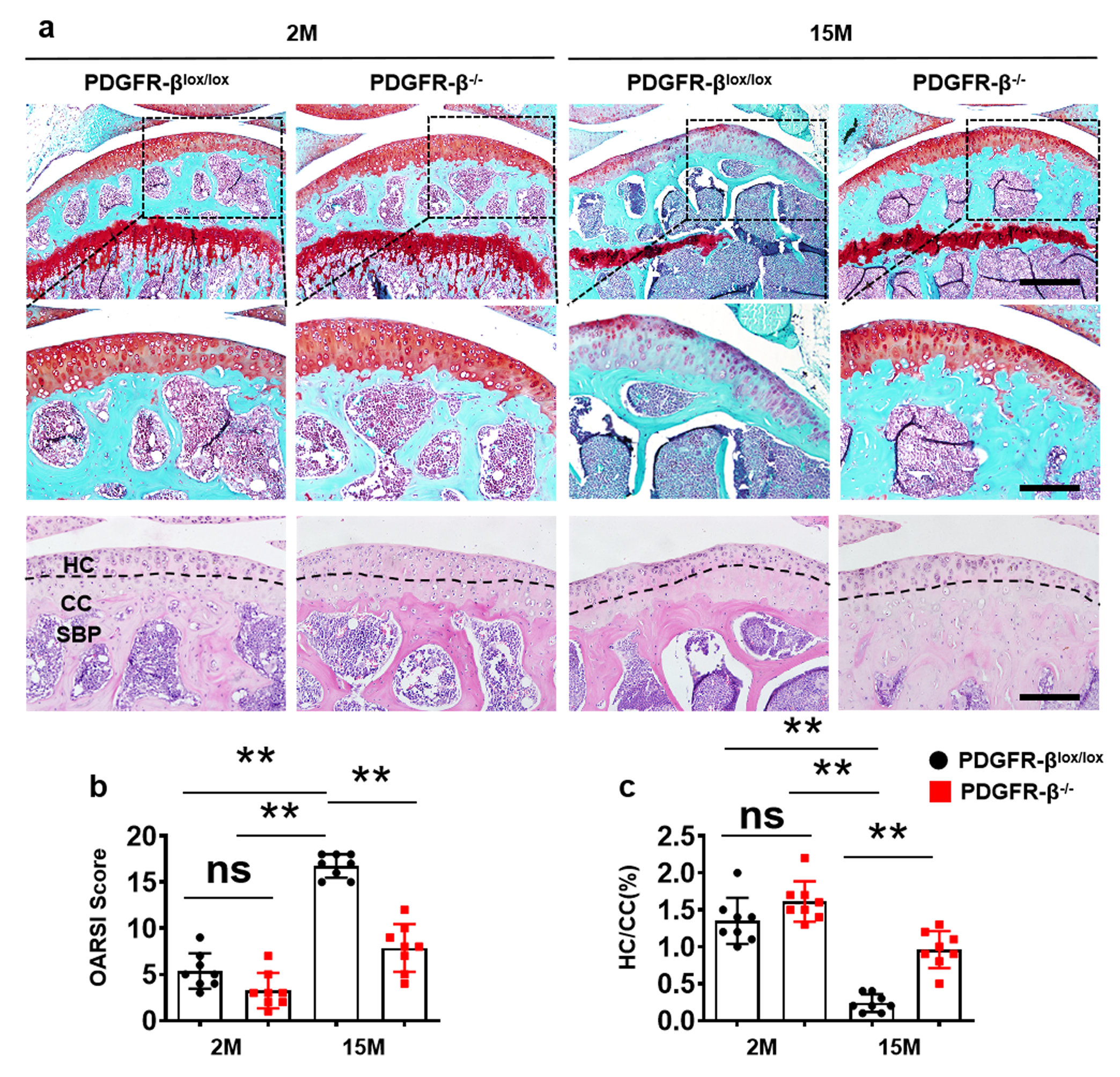

Supplement: Supplementary file 7 — Supplementary figure 3 [file 41413_2022_229_MOESM7_ESM.tif]

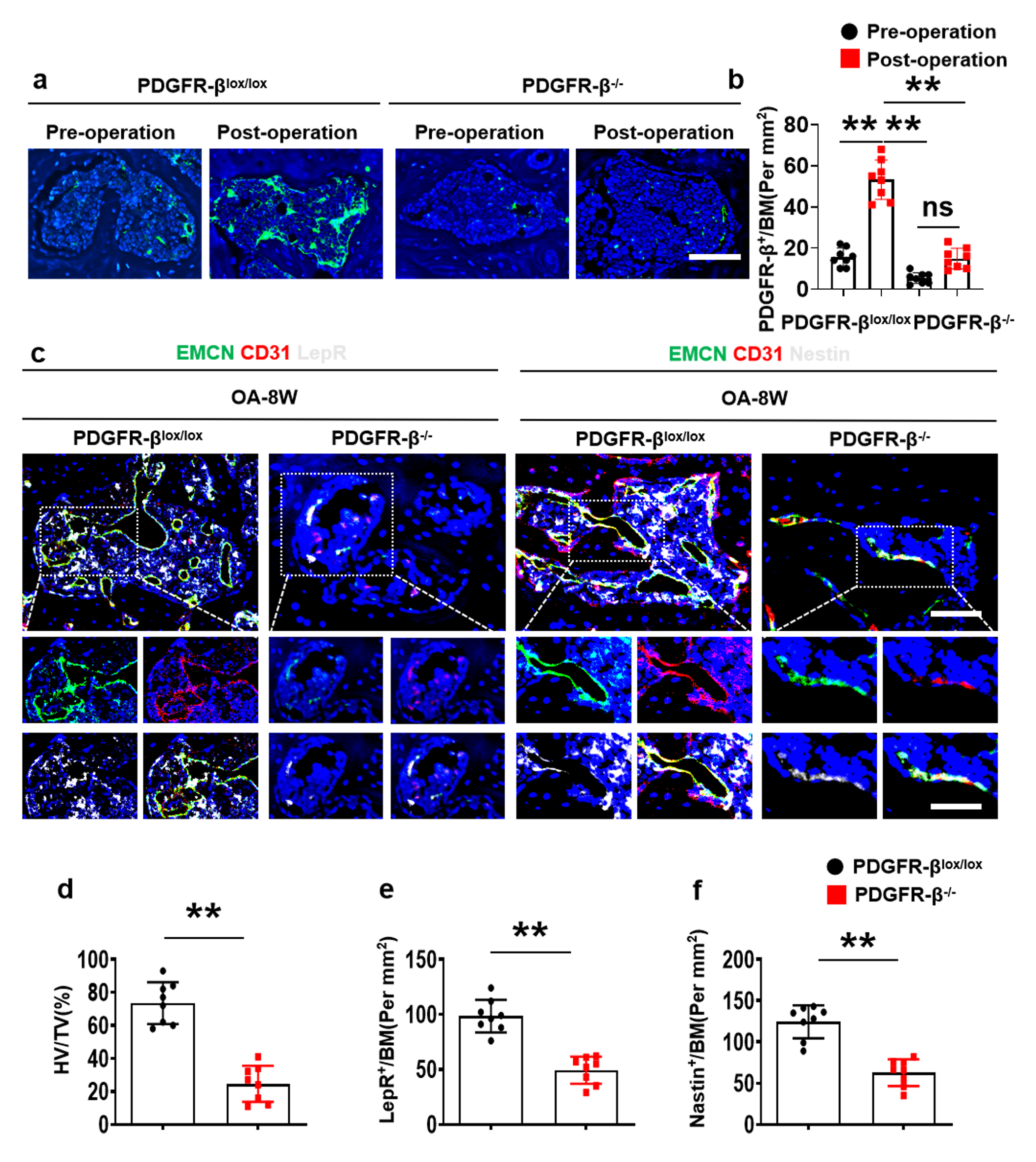

Supplement: Supplementary file 8 — supplementary figure4 [file 41413_2022_229_MOESM8_ESM.tif]

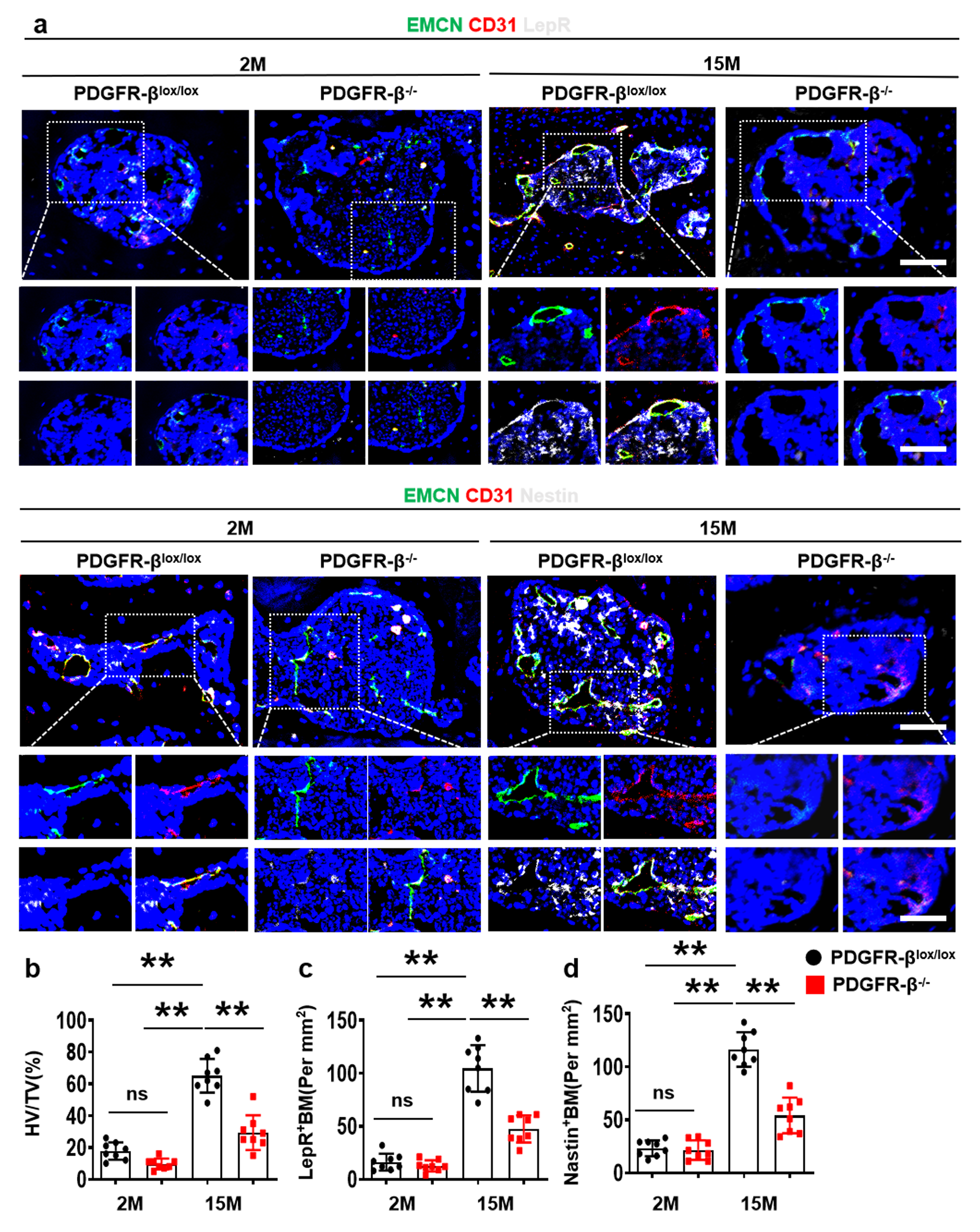

Supplement: Supplementary file 9 — supplementary figure5 [file 41413_2022_229_MOESM9_ESM.tif]

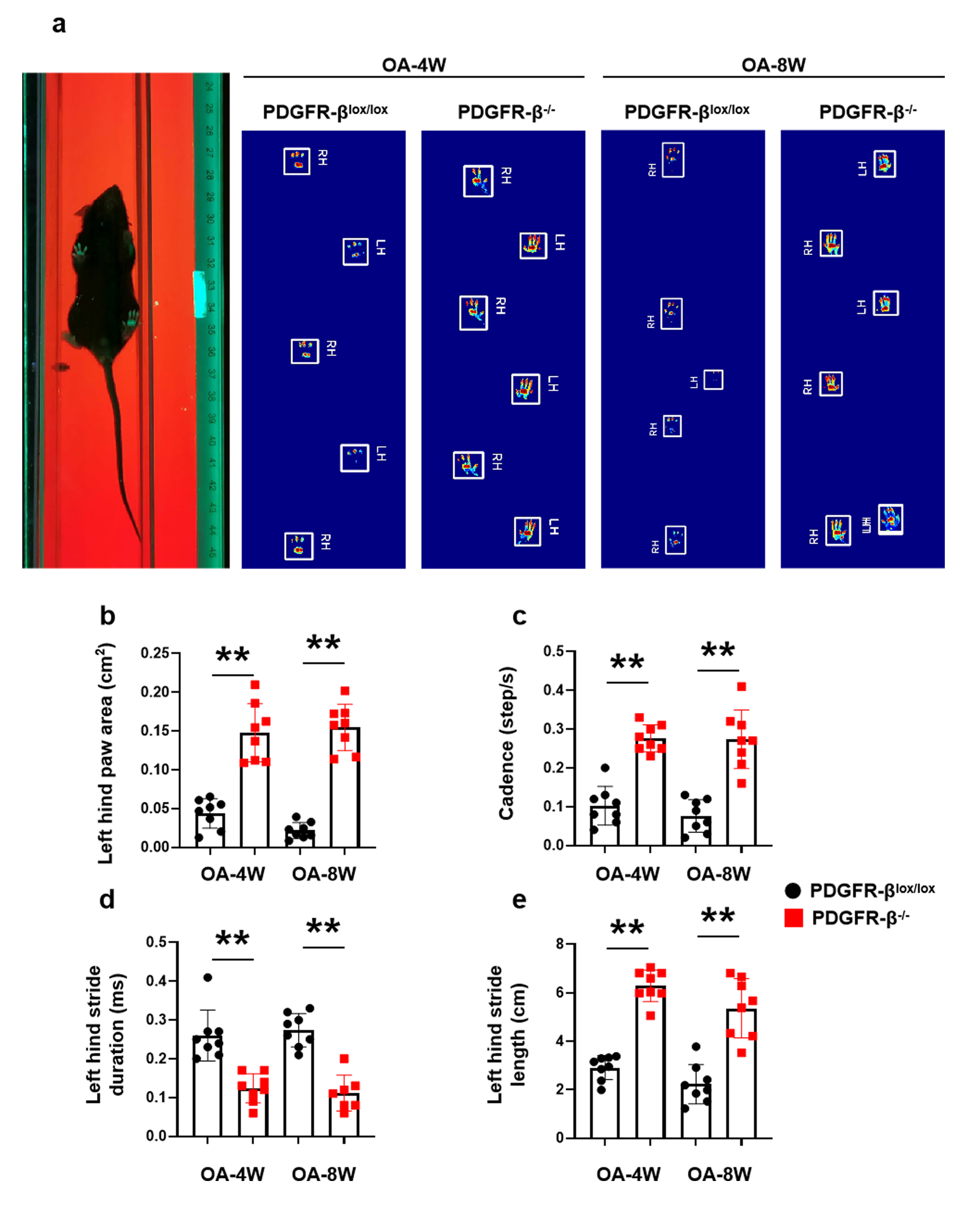

Supplement: Supplementary file 10 — Supplementary figure 6 [file 41413_2022_229_MOESM10_ESM.tif]

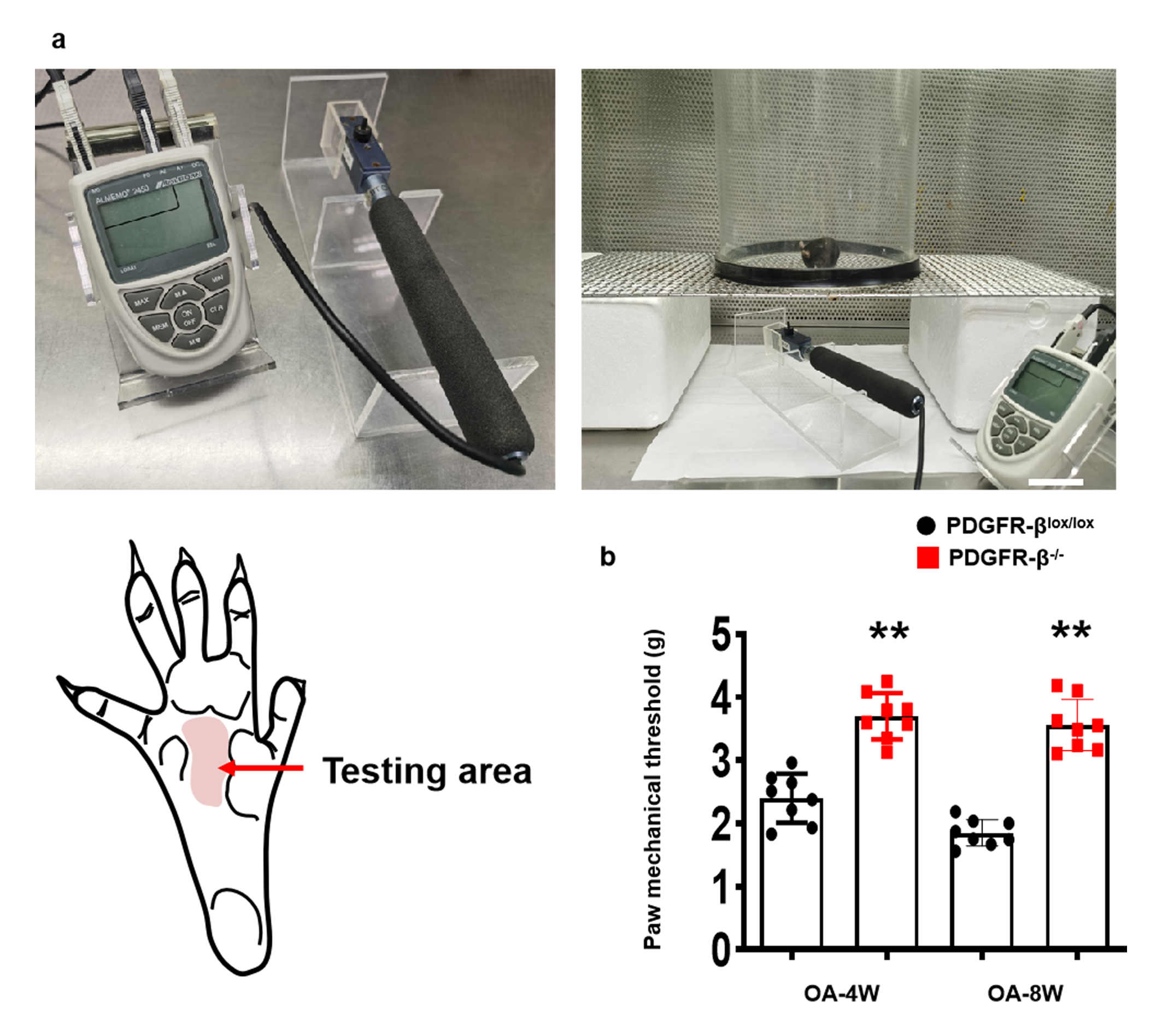

Supplement: Supplementary file 11 — supplementary figure 7 [file 41413_2022_229_MOESM11_ESM.tif]

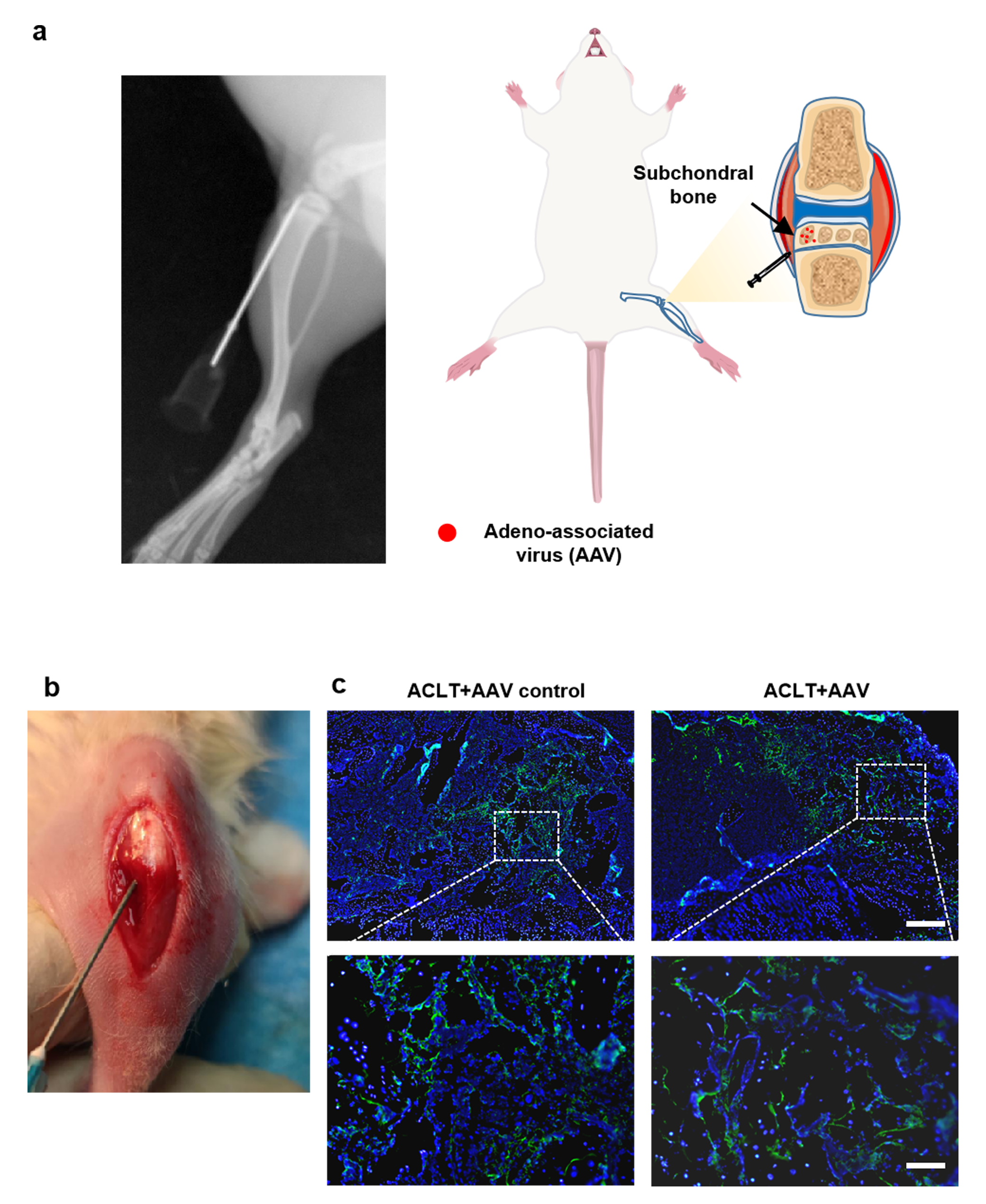

Supplement: Supplementary file 12 — Supplementary figure 8 [file 41413_2022_229_MOESM12_ESM.tif]

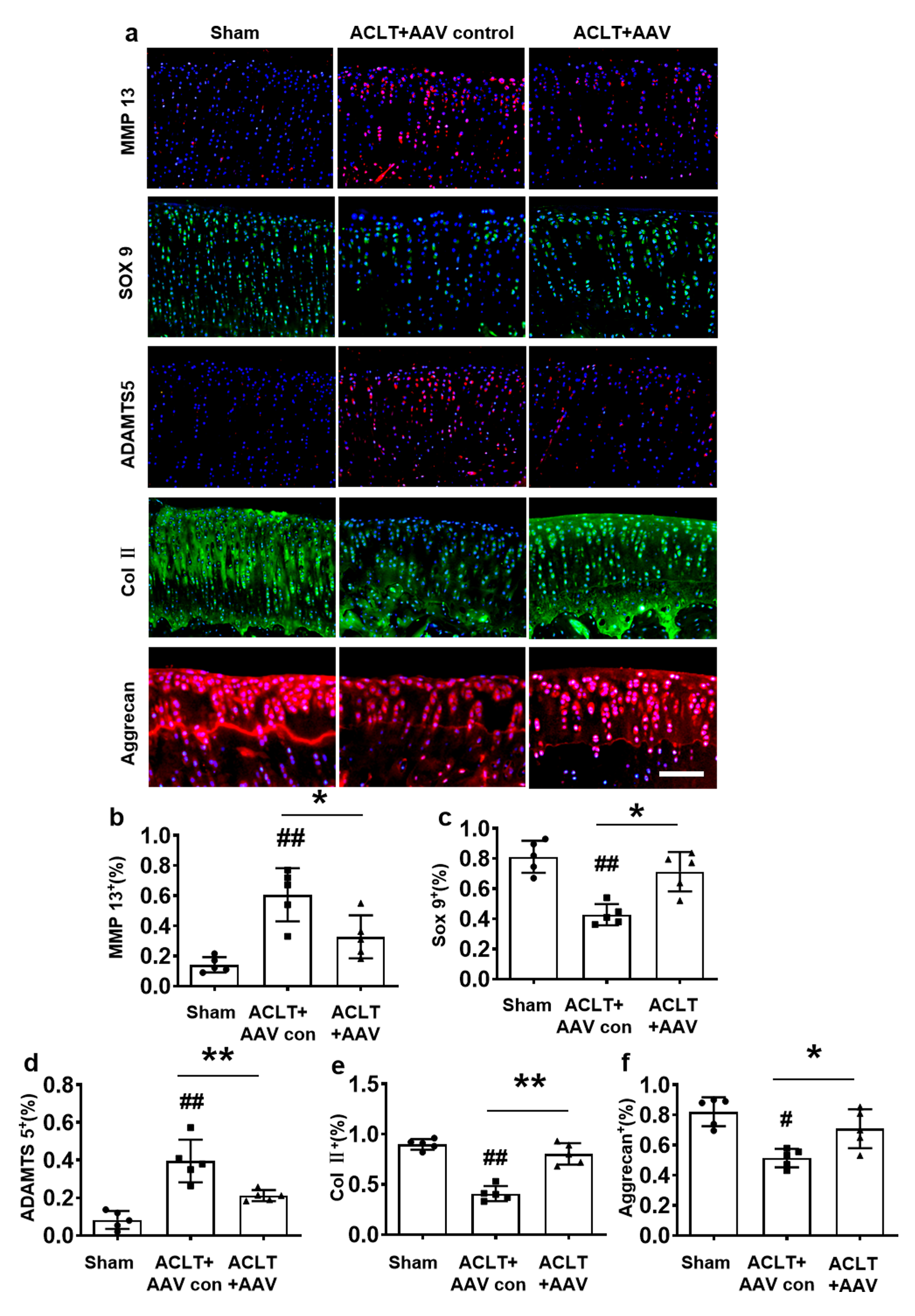

Supplement: Supplementary file 13 — Supplementary figure 9 [file 41413_2022_229_MOESM13_ESM.tif]

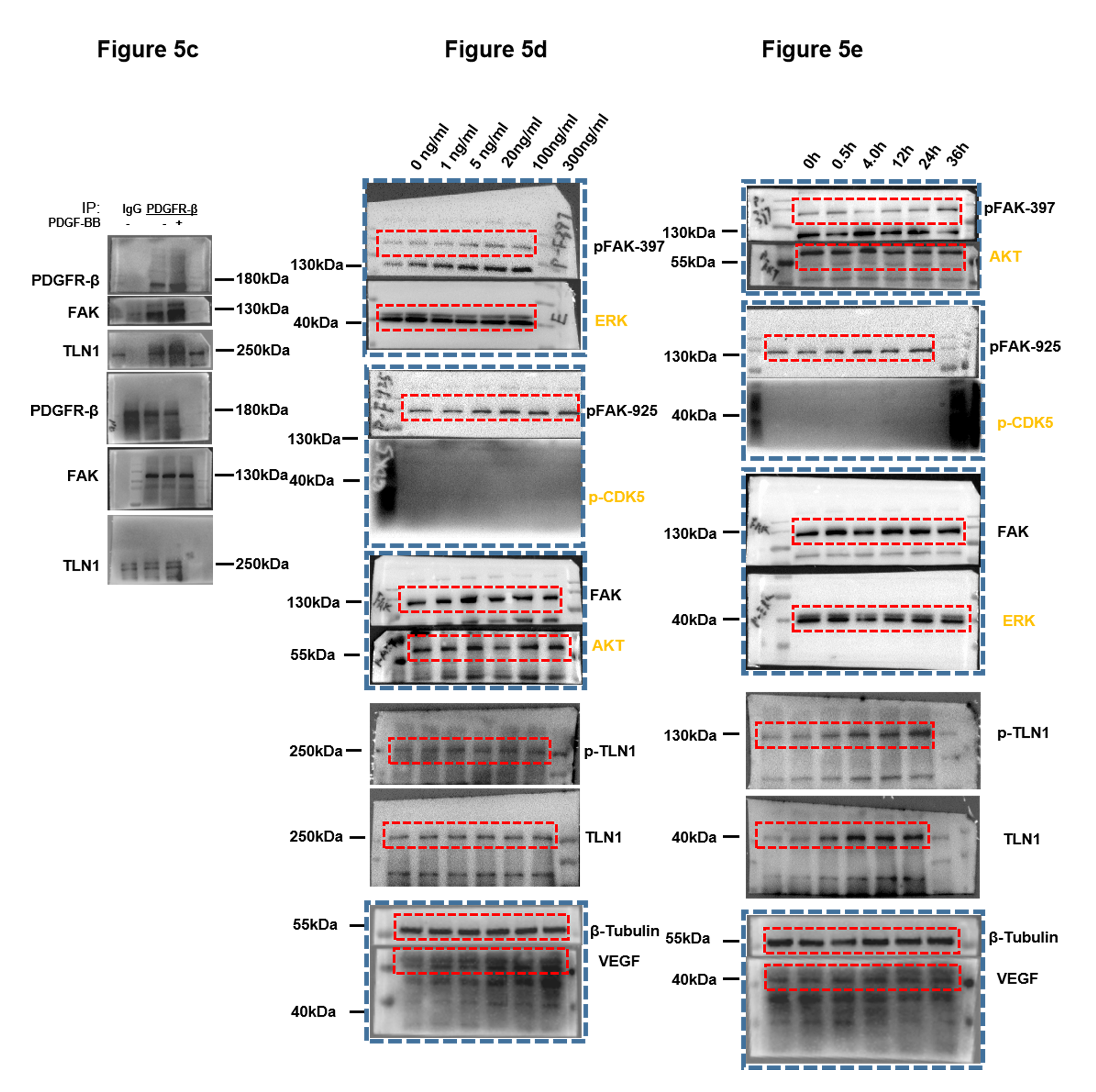

Supplement: Supplementary file 14 — supplementary figure 10 [file 41413_2022_229_MOESM14_ESM.tif]

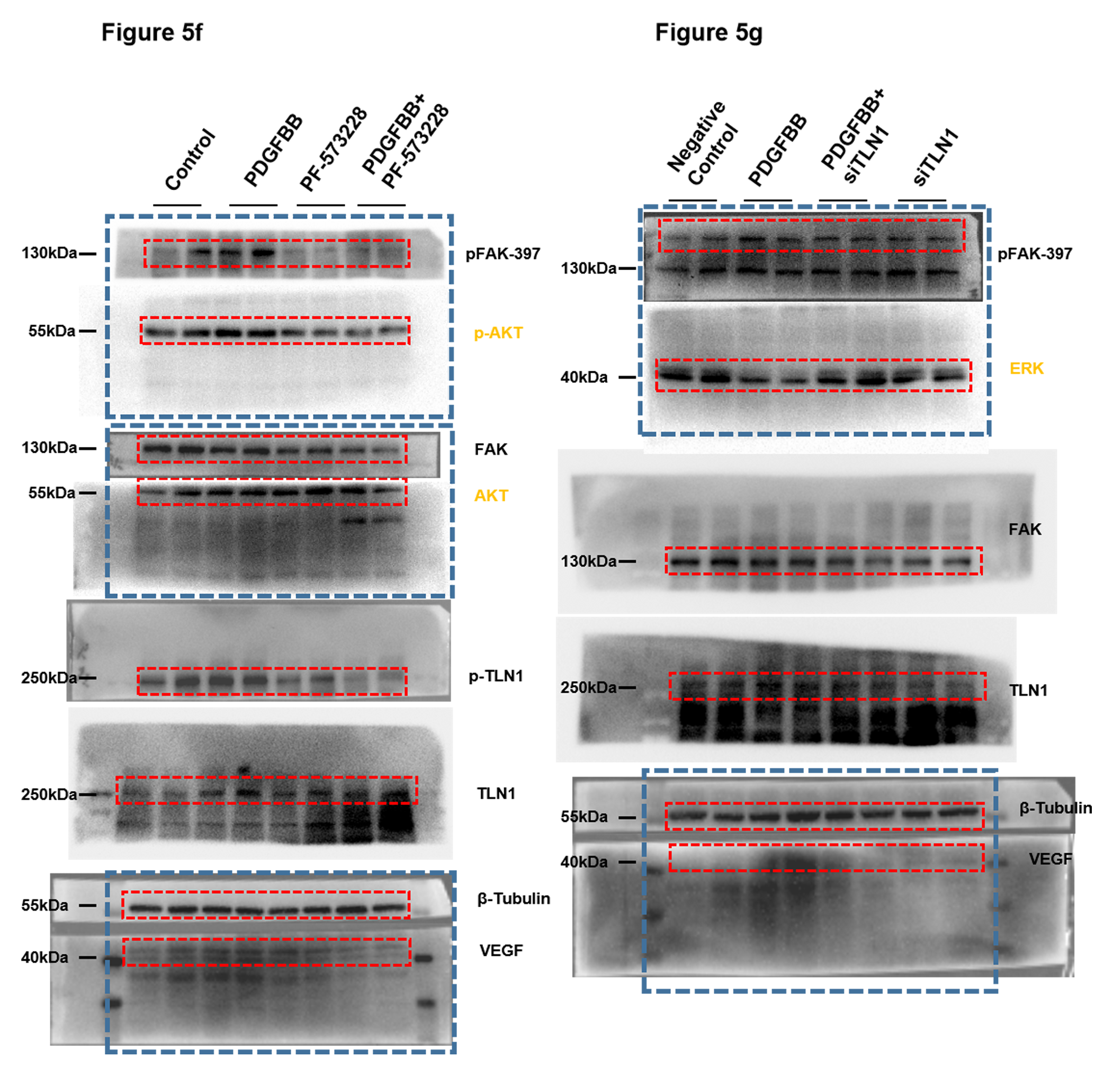

Supplement: Supplementary file 15 — supplementary figure 11 [file 41413_2022_229_MOESM15_ESM.tif]
